# Parental influence on begging call structure in zebra finches (*Taeniopygia guttata*): evidence of early vocal plasticity

**DOI:** 10.1098/rsos.150497

**Published:** 2015-11-25

**Authors:** Avelyne S. Villain, Ingrid C. A. Boucaud, Colette Bouchut, Clémentine Vignal

**Affiliations:** Université de Lyon/Saint-Etienne, Neuro-PSI/ENES CNRS UMR 9197, France

**Keywords:** songbird, nestling, parent–offspring communication, vocal production learning, cross-fostering, sex differences

## Abstract

Begging calls are signals of need used by young birds to elicit care from adults. Different theoretical frameworks have been proposed to understand this parent–offspring communication. But relationships between parental response and begging intensity, or between begging characteristics and proxies of a young’s need remain puzzling. Few studies have considered the adjustment of nestling begging features to previous experience as a possible explanation of these discrepancies. In this study, we tested the effect of a heterospecific rearing environment on individual developmental trajectories of the acoustic structure of nestling begging calls. Fifty-two zebra finch chicks were fostered either to Bengalese finch or to zebra finch parents, and begging calls were recorded at several stages of nestling development. Acoustic analyses revealed that the development of the spectral features of the begging calls differed between experimental conditions: chicks reared by Bengalese finches produced higher pitched and less broadband begging calls than chicks reared by conspecific parents. Differences were stronger in males than females and were not explained by differences in growth rate. We conclude that nestling begging calls can be plastic in response to social interactions with parents.

## 1. Introduction

Young mammals and birds solicit care from parents using complex begging behaviours [1]. Begging signals are conspicuous and intense performances that involve visual and acoustic cues [2]. Several theoretical frameworks have been used to understand the design of begging signals. Parent–offspring conflict theory suggests that begging behaviour is the result of an evolutionary conflict of interests over resource allocation [3,4]. From this perspective, begging displays are signals by which offspring manipulate parents to provide more care than would be optimal [1] or through which siblings compete for a larger share of parental care [5]. Alternatively, begging behaviours are considered by honest signalling models to be costly [6] and as such, to be reliable signals of needs that allow parents to make decisions on the appropriate amount of care needed by their offspring [7,8]. Accordingly, the intensity of begging by bird nestlings increases with their level of hunger and can stimulate parental feeding [9–13]. However, inconsistencies in the impact of begging intensity on parental response have been puzzling [14,15], and relationships between begging characteristics and several proxies of nestlings’ needs seem complex [12,16–18].

Some of the discrepancies between models and measurements of begging’s correlates might be because of the dynamic of parent–offspring communication. Environmental conditions and learning processes might change the value of a signal on the receiver side and might shape the characteristics of a signal on the emitter side. Parents, on the one hand, may learn to ignore or pay particular attention to some aspects of their offspring’s begging. Parents show varied levels of sensitivity to offsprings’ signals, both between and within species [7,19]. Nestlings, on the other hand, may learn from their previous experience which aspects of their begging display were more rewarding and change their begging behaviour accordingly [16,18,20,21]. Nestlings show a preference for particular positions in the nest previously associated with food provisioning [22,23] and are able to learn to beg at the most rewarding begging intensity [16,24]. This behavioural adjustment in response to experience could allow nestlings to cope with changing conditions, such as variations in parental provisioning or sibling competition and would modify offspring signalling of needs to the parents. Getting a better estimation of the extent of learning in begging is thus important to further our understanding of the evolution of parent–offspring communication.

Surprisingly, learning in nestling begging calls has rarely been investigated. Begging calls are known to carry multiple pieces of information such as individual signature [25] or information about sex [26], hunger level [20,21] or thermal state of the individual [13]. A change in the acoustic structure of begging calls in response to experience has been examined in the particular context of host–parasite interactions. In brood parasitic bird species like the Horsfield’s bronze-cuckoo (*Chalcites basalis*) [27], the common cuckoo (*Cuculus canorus*) [28] or the African indigobirds (*Vidua* species) [29], chicks mimic the begging call structure of their host, and this structural change seems to be shaped by the behavioural response of host parents. In this recognition arms race, one host species (the superb fairy wren, *Malurus cyaneus*) has been shown to use a parent-specific password: females call to their eggs at the end of incubation, and upon hatching nestlings produced begging calls with high similarity to their mother’s call [30]. Parasitic nestlings of the brown-headed cowbird (*Molothrus ater*) exaggerate the host’s display and thus increase food provisioning by host parents. In response to nest parasites, the host nestlings of song sparrow (*Melospiza melodia*) change their begging call to match the parasite [31]. Apart from these examples in host–parasite systems, one study in tree swallow nestlings (*Tachycineta bicolor*) suggested that brood signature in begging calls just before fledging results from interactions between environment and genetic/maternal effects [32]. However, to our knowledge, no study has tracked individual ontogeny of begging call structure to demonstrate vocal adjustment in response to social experience.

In this study, we tested the possibility of learning of the begging calls of male and female nestling zebra finches (*Taeniopygia guttata*). To do so, nestlings were all cross-fostered to control for genetic effects, and raised either by zebra finch parents or heterospecific parents, Bengalese finches (*Lonchura striata* var. *domestica*), which are classically used as social parents in cross-fostering experiments [33]. We recorded begging calls at several stages of nestling development. As chicks get older and heavier, begging calls are expected to shift to lower values of the frequency spectrum [34,35] as well as to increase their spectral bandwidth and duration [35]. If chicks adjust their begging call structure in response to the cross-fostering condition, we expect deviations from this normal developmental trend and differences between cross-fostering groups at a given age. On the one hand, parental feeding could act as a conditioning process [24,36] and chicks could hone in on the acoustic cues that get the greatest response from the parents [28,37]. In this case, we expect zebra finch chicks reared by Bengalese finch parents to call at higher frequencies and with reduced spectral bandwidth, which are acoustic cues of Bengalese finch begging calls and vocal repertoire [38]. On the other hand, the cross-fostering treatment could represent a developmental stress. If chicks reared by Bengalese finch parents are less fed and thus hungrier, begging calls are expected to be longer, produced at a higher call rate [13,34,39] and with more spectral noise [40]. Hungrier chicks may also be more stressed, and from motivation-structural rules [41] and work on adult zebra finches [42], their calls are predicted to be at higher frequencies. If begging calls differ between cross-fostering conditions because of differences in levels of parental care, this should result in growth differences between experimental groups that can be quantified by measuring weight and tarsus length of the young at the end of the nestling period. As several studies suggested that female zebra finches show higher vulnerability to conditions of restricted food [43–45] this hypothesis suggests that females should be more affected than males by the cross-fostering treatment.

## 2. Material and methods

### 2.1 Subjects and housing conditions

Fifty-two chicks of zebra finches (*Taeniopygia guttata*) were used in this study. Zebra finch chicks were produced by 20 parental pairs and were all fostered to male–female pairs of either zebra finches (*N*=12 pairs) or Bengalese finches (*Lonchura striata* var. *domestica*; *N*=8 pairs).

All adult zebra finches came from our breeding colony (ENES Laboratory, University of Saint-Etienne), whereas adult Bengalese finches were purchased from commercial providers, seven weeks before the beginning of the experiments. All pairs were freely formed in aviaries, were between 1 and 2 years old and had previous breeding experience. Pairs were housed separately in breeding cages (80×40×40 cm) equipped with perches, a nest-box and a pool for environmental enrichment. All the birds were kept under the same environmental conditions (temperature between 24°C and 26°C, light conditions 14 L:10 D h). Birds were fed finch seed cocktails, egg paste, water and cuttlefish bones *ad libitum* and supplemented with salad once a week.

### 2.2 Cross-fostering procedure and experimental groups

Chicks were cross-fostered at 2 or 3 days old by transferring them from their parental nest to the nest of their foster parents. Foster parents were either zebra finches (chicks reared by ZF=ZFR) or Bengalese finches (chicks reared by BF=BFR). No chick was reared by its genetic parents and genetic siblings were split between different foster families: cross-fostered social broods thus included chicks from different genetic parents. Ages of the chicks in an experimental brood differed from 0 to 4 days, as can be observed in the wild [46]. To avoid an effect of brood size on development [47], experimental brood size was fixed at three chicks per nest, but depending on chicks hatching date and synchrony between nests of origin, it was sometimes not possible to cross-foster three chicks in all foster nests, so some pairs reared only two chicks (see brood composition, electronic supplementary material, table S2). Experimental groups were similar in brood size (mean±s.d.: 2.82±0.6 and 3.13±0.35 for ZFR (16 broods) and BFR (eight broods) groups, respectively, Wilcoxon rank sum test, *W*=32, *p*=0.2189) and in chicks’ mortality (21.8% in BFR, 20.33% in ZFR, $\chi_{,1}^{2}<0.001,p=0.92$).

The two experimental groups (zebra finch parents or Bengalese finch parents) were housed in separate and acoustically isolated rooms. So the chicks’ auditory experience was restricted to their experimental condition.

Chicks’ sex was determined by molecular sexing (Genindexe, http://www.genindexe.com/) using feather samples. The intra-brood sex-ratio was calculated as relative proportion of females and males in the brood (electronic supplementary material, table S2). Sex-ratio did not differ between the two groups (mean±s.d.: 0.59±0.19 and 0.51±0.19 for ZFR (16 broods) and BFR (eight broods) groups, respectively, Wilcoxon rank sum test, *W*=51.5, *p*=0.5406).

The overall sex composition of the groups was as follows:

(1) BFR: 12 females and 10 males.
(2) ZFR: 15 females and 13 males.

### 2.3 Analysis of begging call development

#### 2.3.1 Data collection

All chicks were recorded at three different developmental stages (6±1, 9±1, 14±1 days post-hatching (DPH)) after removing the chick from the nest temporarily.

Because begging call structure strongly depends on the level of hunger of the chick [40], we controlled for chicks’ hunger level at the time of recording by recording them at their maximum level of motivation. To do so, we submitted the chicks to a short food deprivation during which they remained in the nest in the breeding cage, but the access to the nest was blocked for the parents. We increased the time of deprivation (60 min at 6 DPH, 90 min at 9 DPH, 120 min at 14 DPH), mimicking the natural inter-visit interval of the parents [46]. We also checked that only a few seeds remained in the chick’s crop (visible through the skin) before the recording. To induce begging calls from the nestlings, we used beak stimulations with a small red stick that mimicked an adult beak. Until 9 DPH, the chick’s eyes are closed, so begging calls were predominantly triggered by the tactile stimulation. After this age, stick stimulation was still equally efficient in both experimental groups. After 9 DPH, 4297 calls were recorded in BFR and 4352 in ZFR.

Each chick was placed in an experimental nest furnished with cotton located in a room adjacent to the breeding colony and recorded using an Audio Technica 803 tie-microphone placed at 10 cm from the chick and connected to a Marantz PMD 671 recorder.

#### 2.3.2 Acoustic analysis

Depending on individuals, 10–50 begging bouts (each bout being a chain of several repeated calls, on average 4.43±4.24 per individual) were extracted at 6, 9 and 14 DPH. A total of 10 621 calls (5364 in BFR, 5653 in ZFR) were extracted and analysed.

Acoustic analysis was performed using custom-written codes using the Seewave package [48] implemented in R [49]. After bandpass filtering (250–15 000 Hz, Seewave ‘fir’ function) and intensity normalization, the call duration was measured via the Seewave ‘timer’ function and the following spectral parameters were computed using the Seewave ‘specprop’ function (FFT using a Hamming window and a window length of 512):

— The mean, the median, the first (=Q25) and third (=Q75) quartiles, the inter-quartile range (=IQR), the standard deviation (=s.d.) and the mode of the call frequency spectrum (all in Hertz).
— The spectral flatness (=Sfm) of the frequency spectrum—a measure of the signal’s noisiness. Sfm of a noisy signal tends towards 1, whereas Sfm of a pure tone tends towards 0.
— The skewness—a measure of the distribution symmetry of the frequency spectrum of the call. Skewness tends to 0 when the spectrum is symmetric, is positive when the spectrum is skewed to right and negative when the spectrum is skewed to left.

#### 2.3.3 Statistical analysis

All statistical analyses were performed using R software [50]. Because the dataset contained missing values, statistics were computed on 9423 calls (4273 in ZFR and 5150 in BFR), from 51 chicks. Electronic supplementary material, table S3 gives the number of calls per session for each subject of each group.

We first performed a principal component analysis (PCA) on acoustic parameters of the calls (1), and then built a linear mixed effects model to test the effect of the experimental treatment on the principal components (PCs) resulting from the PCA (2). The effect of the experimental treatment on each parameter separately is presented in the electronic supplementary material. Finally, we tested the effect of the experimental treatment on chicks’ body condition using a linear mixed model (3). All models were validated by checking residuals’ equivariance and symmetrical distribution. Model stability was checked using a custom-written function, written by Roger Mundry.

*PCA on acoustic parameters*. PCA is commonly used in behavioural analysis to reduce the number of variables by eliminating redundancy caused by intercorrelation and emphasizes mutual dependencies between them. It is particularly useful because subjects can display different combinations of behaviours to express the same functional response [51]. Before the PCA, parameters with non-symmetrical distributions were transformed. The PCA was performed on 10 acoustic parameters (‘dudi.pca’ function of ‘ade4’ R package): mean, s.d., median, mode, Q25, Q75, skewness, Sfm, call duration (for variable composition, see table 1). The two first PCs of the PCA, which had eigenvalues above one, were kept.

**Table 1.** Variable compositions of the PCA on acoustic parameters. Transformations are indicated in parentheses. Percentage of each parameter composing the PC,^a^ percentage of explained variance and eigenvalues of each PC are indicated.

	PC1	PC2
explained variance (% cumulative)	*50*.*7*	*79*.*6*
eigenvalue	*5*.*1*	*2*.*9*
mean	−18.65	−0.03
s.d.	−0.08	31.37
median	−17.9	−1.01
mode	−9.48	−8.29
Q25	−14.1	−7.05
Q75	−16.41	3.83
IQR	−1.88	28.79
Sfm	−6.89	16.66
skewness (square root)	10.43	0
call duration (log)	−4.17	−2.97

^a^Absolute contributions of the decomposition of inertia for each PC (‘inertia.dudi’ function from ‘ade4’ R package), divided by 100 to get the percentage. Signs are the signs of the coordinate.

*Linear mixed effect models on principal components.* Each PC was analysed using a linear mixed effects model (‘lmer’ function from ‘lme4’ R package [52]). We built a model including fixed and random factors having a potential effect on call structure considering the design of the experiment. To increase the interpretability of the results, all continuous variables included in the model were *z*-transformed [53] (indicated with a ‘*z*’ before the factor’s name in the model formula).

The following model was computed:

model <- lmer(PC ∼ Group *zAge* Sex+zcall index+zsex-ratio+zBCI+(1|Subject)+(0+zAge|Subject)+(1|Nest of Origin)+(0+ SexF|Nest of Origin)+(0+GroupFR|Nest of Origin)+(0+zAge|Nest of Origin)+(0+SexF: zAge|Nest of Origin)+(1|Foster Nest)+(0+sexF|Foster Nest)+(0+zAge|Foster Nest)+(0+SexF: GroupFR|Foster Nest)+(0+SexF:zAge|Foster Nest)+(1|sessionID).

‘Group’, ‘Sex’ and ‘Age’ and their interactions were used as fixed factors to test (i) the effect of the treatment throughout chicks’ development and (ii) potential differential effects in male and female chicks. Because ‘Age’ was analysed as a continuous covariate, the effect of the cross-fostering treatment was not analysed for each age point separately but on changes of call acoustic structure over the development. During the recording session, the motivation to beg of the chicks could change due to the absence of food. We thus included the call index in the sequence (‘call index’) as an additional fixed factor. Intra-brood sex-ratio (‘sex-ratio’) or chicks’ body condition index (‘BCI’, see §2.4) could also have had an effect on call acoustic structure and were added as fixed factors as well. Because our experimental design used repeated recordings of calls per subject and per age, two random factors were added. First, we used a ‘sessionID’ random factor, which is a unique term of the session of recording for a given chick at a given age. Second, a ‘subject’ random factor was used to deal with repeated measures on each chick. As recommended for within-subjects design with a covariate [51], ‘Age’ was also included as a random slope within the subject. To control for genetic and social effects on call structure, the ‘Nest of Origin’ and the ‘Foster Nest’ were added as random factors. Again, as recommended for within-subjects design with a covariate [51], experimental group (‘GroupFR’) and sex (‘SexF’) were included as random slopes after being manually dummy coded (binary code: 1 if true, 0 if false). Interactions between random slopes were considered only when the sample size in each corresponding subset of data was sufficient.

*P*-values were assessed using the ‘drop1’ function (‘stats’ R package). ‘Drop1’ computes likelihood ratio test statistics and *P*-values for all single terms, fits those models and computes a table of the changes in fit. Only relevant interactions then compose the statistical table. When the three-way interaction was significant (Group: Sex: zAge), post hoc models were run either in each sex separately or in two categories of age (old nestlings: Age≥12 or young nestlings: Age≤7). Estimates of fixed factors for each model were computed with the ‘lsmeans’ function (‘lmerTest’ R package) and are available in the electronic supplementary material.

### 2.4 Analysis of chicks’ body condition

#### 2.4.1 Data collection

To control potential effects of cross-fostering treatment on chicks’ body condition, two morphological features of the chicks were measured once at 14 (±1) DPH. Tarsus and weight were measured using a calliper (±0.1 mm) and a Pesola scale (±0.1 g), respectively. All measures were done at the same time of the day (noon), after acoustic recordings.

We calculated the BCI as residuals of the linear regression model between tarsus length and weight at day 14 (±1).

All of the 52 nestlings were measured, but only 23 of them were recorded at 14 (±1) DPH. Results of the BCI analyses on the complete dataset (52 nestlings) or on the subset of data restricted to the nestlings recorded at 14 (±1) DPH (23 nestlings) did not differ, so only the first is presented.

#### 2.4.2 Statistical analysis

To test the effect of the treatment and take into account the fact that male and female zebra finches can have initial differences in growth rate [43], we used the following linear model:

modelBCI <- lmer(BCI values ∼Group*Sex+Age at measurement+zsex-ratio++(0+ groupFR|Foster Nest)+(0+GroupFR|Nest of Origin)+(0+sexF|Foster Nest)+(0+sexF|Nest ofOrigin).

‘Group’, ‘Sex’ and their interaction were included as fixed factors. The ‘Age at measurement’ (unique per chick) was added to control the effect of between-individuals variation in age at measurement (14±1). The intra-brood sex-ratio (‘sex-ratio’) was added to control the effect of brood composition on chick body condition and this continuous parameter was *z*-transformed. The Nest of Origin and the Foster Nest were used as random factors and dummy coded Sex and Group were used as random slopes.

*P*-values were assessed using the ‘drop1’ function. Estimates of fixed factors for each model were computed with the ‘lsmeans’ function and are available in the electronic supplementary material.

The model was validated by checking residuals’ equivariance and symmetrical distribution (‘plotresid’ function from ‘RVAideMemoir’ R package). Model stability was checked using a custom-written function, written by Roger Mundry.

## 3. Results

### 3.1 Cross-fostering to heterospecific parents differentially affected the development of male and female begging calls

The cross-fostering treatment affected the structure of begging calls produced by nestlings. PC1 (explaining 50.7% of the total variance; table 1) was significantly affected by the triple interaction between group, age and sex of the chicks but not PC2 (explaining 28.9% of the total variance; table 2; electronic supplementary material, table S4a). Post hoc tests were run on PC1: (i) in males and females separately and (ii) in young and old nestlings separately.

**Table 2.** Statistical results from the ‘drop1’ function (‘lmerTest’ R package) computed on the full model (*a*) and post hoc models following significant interactions(*b*).

	d.f.	LRT	Pr(Chi)
(*a*) results from ‘drop1’ function on all data			
*PC1—all data*			
call number	1	0.356	0.551
intra-brood sex-ratio	1	0.393	0.531
BCI	1	0.000	0.993
Social Group:Sex:Age	1	10.529	*0*.*001*
*PC2—all data*			
call number	1	65.212	*0*.*000*
intra-brood sex-ratio	1	2.810	0.094
BCI	1	0.001	0.972
Social Group:Sex:Age	1	2.866	0.090
(*b*) results from ‘drop1’ function on post hoc models on PC1			
(i) analysis of the Social Group: Age interaction			
*PC1—post hoc test on males*			
call number	1	4.432	*0*.*035*
intra-brood sex-ratio	1	0.019	0.889
BCI	1	0.034	0.853
Social Group: Age	1	4.399	*0*.*036*
*PC1—post hoc test on females*			
call number	1	3.383	0.066
intra-brood sex-ratio	1	0.939	0.332
BCI	1	0.096	0.757
Social Group: Age	1	1.609	0.205
(ii) analysis of the Social Group: Sex interaction			
*PC1—post hoc test on young nestlings*			
call number	1	0.000	0.989
intra-brood sex-ratio	1	0.286	0.593
BCI	1	0.126	0.723
Social Group: Sex	1	0.253	0.615
*PC1—post hoc test on old nestlings*			
call number	1	0.000	0.995
intra-brood sex-ratio	1	0.139	0.709
BCI	1	0.062	0.804
Social Group: Sex	1	7.056	*0*.*008*
*PC1—post hoc test on old male nestlings*			
Social Group	1	10.545	*0*.*001*
call number	1	2.333	0.127
intra-brood sex-ratio	1	0.741	0.389
BCI	1	0.009	0.926
*PC1—post hoc test on old female nestlings*			
Social Group	1	0.028	0.868
call number	1	4.473	0.034
intra-brood sex-ratio	1	0.082	0.775
BCI	1	0.015	0.903

**Figure 1.**
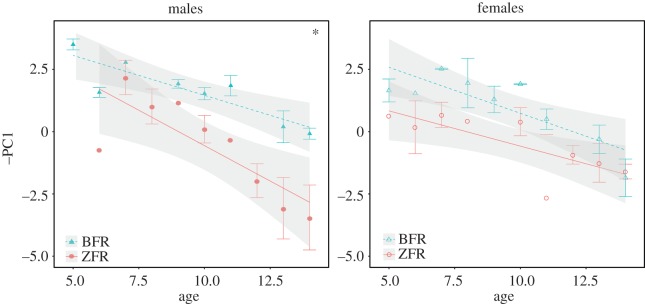
Modifications of begging call acoustic structure over the development of zebra finches reared either by zebra finches (ZFR, *N*=24, 13 females and 11 males) or by Bengalese finches (BFR, *N*=21, 12 females and nine males). PC1 is the first principal component from a PCA on acoustic parameters. Points are mean (±s.e.) when the corresponding subset of data contains several chicks, and mean only (without error bar) when the data point represents only one chick. The dataset for one chick at a given age still represents multiple calls, so the corresponding data point is the mean of all these calls. Grey shades are 95% CIs of the linear regression. Because the three-way interaction was significant (cross-fostering group: sex: age; table 1), post hoc tests were performed for each sex. Significance of the interaction between cross-fostering group and age as a covariate is indicated in insert: *p≤0.05.

The interaction between group and age was significant in males but not in females (figure 1 and table 2; electronic supplementary material, table S4b). The call spectrum shifted to lower frequencies during development (increasing PC1 values). But this developmental shift was influenced by the cross-fostering group in males only: it was slower in BFR chicks than in ZFR chicks. Thus, male chicks fostered to Bengalese finch parents produced calls with more energy in higher frequency bands later in development than control chicks (figure 3; and electronic supplementary material, figure S3).

**Figure 2.**
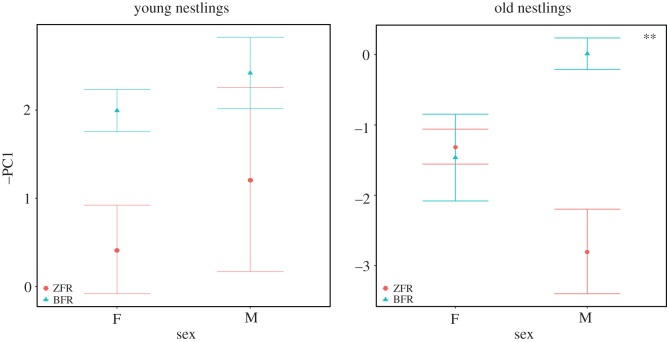
Modifications of begging call acoustic structure of young nestlings (5≤ Age≤7 DPH) and old nestlings (12≤Age≤14 DPH) fostered either to zebra finch parents (*N*=14, seven males and seven females) or Bengalese finch parents (*N*=15, seven males and eight females). Points are means (±s.e.) of all chicks. When the cross-fostering group: sex interaction was significant, post hoc tests were performed in each sex separately (table 2). In females, post hoc tests were all non-significant; in males, significance is indicated in insert: ^**^p≤0.01.

In young nestlings (5≤Age≤8 DPH), the cross-fostering treatment had no effect on PC1 whereas in old nestlings (12≤Age≤15 DPH), we found a significant interaction between cross-fostering group and sex (figure 2 and table 2; and electronic supplementary material, table S4b). So the effect of the cross-fostering treatment observed in males appeared during development and was more pronounced at the end of the nestling period, whereas female calls were not affected by the treatment.

The call index affected PC2 in both sexes and PC1 in males only (table 2). Calls produced later during the recording session had an up-shifted and less broadband frequency spectrum but this result did not seem to interact with the cross-fostering treatment (see the electronic supplementary material, additional figure S2).

### 3.2 Divergent growth trajectories do not explain begging call structure differences

To determine the effect of the cross-fostering group on begging call structure development, the potential side effects on growth of the cross-fostering to heterospecific parents needed to be controlled for. Bengalese finches could feed their chicks more or less leading to differences in chicks’ physiology and condition that could be responsible for the observed changes in acoustic structure of begging calls. There was no significant difference in BCI at 14 DPH between BFR chicks and ZFR chicks (figure 4 and table 3; electronic supplementary material, table S9).

**Figure 3.**
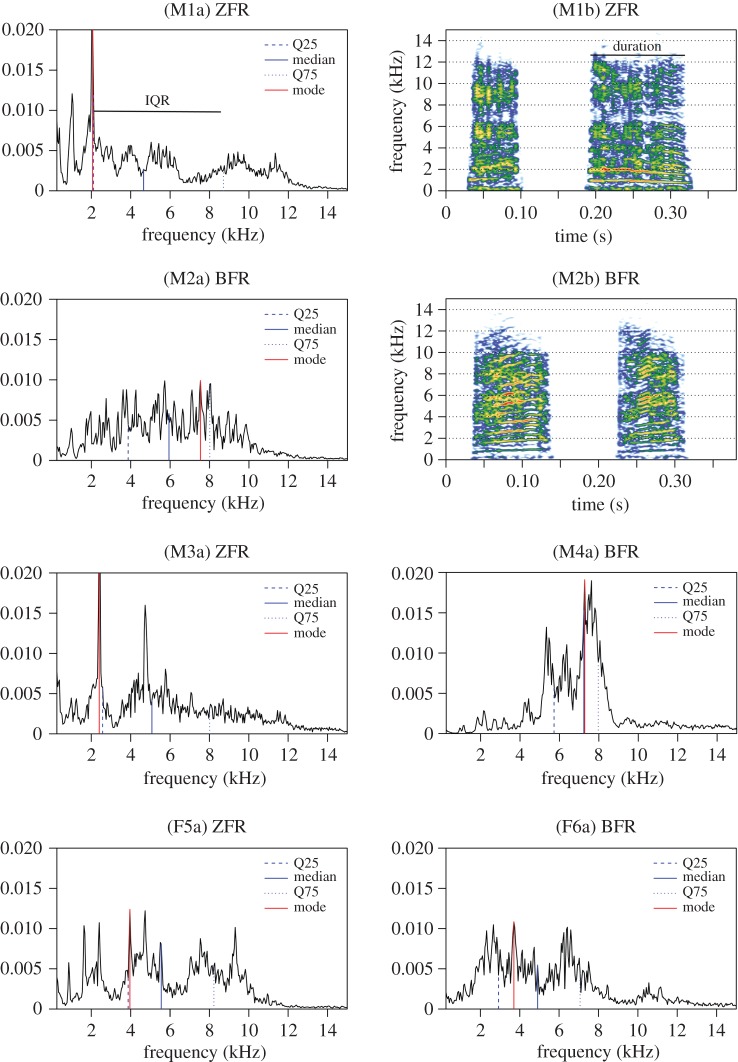
Effect of the cross-fostering treatment on spectro-temporal features of begging calls. Examples of begging calls recorded at 14 DPH on six different subjects (M=males, F=females), either fostered to zebra finch parents (ZFR) or to Bengalese finch parents (BFR). Two representations of the calls are used: ‘a’ labels refer to call spectra with annotations of several spectral parameters (Q25, Q75, median and mode) and ‘b’ labels to spectrograms of begging bouts of two calls with annotation of call duration.

**Figure 4.**
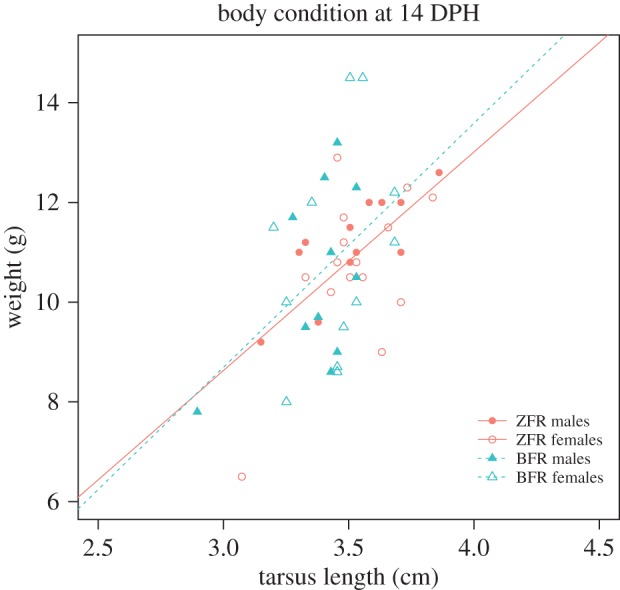
Effect of the cross-fostering group and sex on the BCI of zebra finch chicks fostered either to zebra finch parents (ZFR) or to Bengalese finch parents (BFR).

**Table 3.** Results from the ‘drop1’ function (‘lmerTest’ R package) computed on the full model.

	d.f.	LRT	Pr(Chi)
*BCI—all data (51 subjects)*			
intra-brood sex-ratio	1	0.317	0.573
day of measurement (day 14±1)	1	0.070	0.792
Group: Sex	1	0.404	0.525

## 4. Discussion

In this study, we found that an early cross-fostering event to Bengalese finch parents affected the development of acoustic features of begging calls of young zebra finches. As chicks get older and heavier, begging calls are expected to shift to lower values of the frequency spectrum [34,35], as well as to increase their spectral bandwidth and duration [35]. If chicks adjust their begging call structure to the cross-fostering condition, we expected deviations from this normal developmental trend. Several features of the frequency spectrum differed between BFR chicks and control ZFR chicks. In males only, the normal developmental shift of the frequency spectrum to lower frequencies happened more slowly in BFR chicks. Changes observed in the begging call acoustic features were greater in old nestlings than in young nestlings, confirming an effect of the cross-fostering treatment on the ontogeny of begging calls.

Several hypotheses can be raised to interpret the effect of the heterospecific cross-fostering on begging call ontogeny. A first explanation could be physiological: changes in begging call characteristics could be a side effect of the cross-fostering event on the physiology and the growth rate of the chicks. For example, differences in stress and satiety levels, due to low levels of food provisioning by the parents, could have influenced begging call structure. BFR chicks could have had calls with more energy in high-frequency bands, because they were smaller or hungrier as demonstrated in nestling tree swallows (*Tachycineta bicolor*) or in nestling Jackson’s golden-backed weaver (*Ploceus jacksoni*) [27–29]. But if they were hungrier, their begging calls would have been longer, produced at a higher call rate [13,34,39] and with more spectral noise [40]. We found that call duration and spectral noise (spectral flatness) loaded weakly on PCs, and separate analysis of each parameter (electronic supplementary material, tables S6–S8) showed no effect of the cross-fostering group. Moreover, the two experimental groups did not differ in body condition at 14 days of age, when the acoustic differences between groups were the most pronounced. Finally, several studies have suggested that female zebra finches show higher vulnerability to developmental stress, growth rate and body mass being lower in females than males after food restriction [43–45]. If the cross-fostering to Bengalese finch parents represented a developmental stress, we would expect females to be more affected than males. Females did not differ from males in body condition at 14 DPH, and female begging calls were less affected by the cross-fostering treatment. So differences in acoustic structure of begging calls do not seem to be explained by differences between experimental groups in body condition or in motivation to beg linked to different levels of parental care. Nevertheless, it remains to be investigated whether different patterns of care in the two species could lead to differences in physiology that are not manifested neither in standard measures of body condition nor in our measures of begging acoustic features.

Alternatively, the changes in call structure could be shaped by feeding and brooding responses of the parents: chicks would adjust their begging call structure as a result of the reinforcement they get from food or care delivered by the parents. Bengalese finch parents could be more sensitive to specific cues in the begging call (natural begging call cues of Bengalese finch chicks) and would positively reinforce begging calls that fit these cues. As a consequence, chicks would modify their begging calls by reinforcement learning to resemble Bengalese finch begging calls. Unfortunately, no description of Bengalese finch begging calls is available in the literature. Compared to zebra finch calls, adult Bengalese finch calls show a reduced spectral bandwidth and a higher pitch [38]. A preliminary comparison of Bengalese finch and zebra finch begging calls at 14 DPH (electronic supplementary material, figure S5) shows that the former have a much smaller spectral bandwidth centred around the mean frequency. The spectral noisiness of the signal was also lower. Our results show that BFR nestlings (particularly males) decreased the spectral bandwidth of their calls (lower s.d.) and increased their frequency (higher mean, median, Q25 and Q75). A preliminary comparison of composite scores of the acoustic structure of begging calls from control Bengalese finches, control ZFR zebra finches and BFR zebra finches shows that the latter present intermediate scores between the two control groups (electronic supplementary material, table S10 and figure S6). Taken together, all these results are in favour of the hypothesis that chicks converged with Bengalese finch begging calls. This would be consistent with the reinforcement learning hypothesis, chicks homing in on what stimulates parents best. This mechanism has been described in some cuckoo species [27–29]. Following this hypothesis of begging call structure being shaped by parental response, it is possible that male and female chicks take a different developmental trajectory if they get different food reinforcement. Previous studies demonstrated parental favouritism in zebra finches, male nestlings receiving more food than females [54] especially under poor environmental conditions [55]. Other studies also show that male nestlings beg more strongly during the first days after hatching [56]. If this is the case during cross-fostering to Bengalese finch parents, it means that young males get more occasions to test the efficiency of their begging call structure by trial and error, and thus more occasions to adjust. However, this should translate into sex differences in growth, which we did not observe at the end of the nestling period.

A third possibility is that vocal plasticity could be the result of either social acoustical stimulation or vocal imitation. In the former hypothesis, the global acoustic context could be responsible for the changes in begging call structure by vocal improvization. When hand-reared in groups, Oregon juncos *Junco oreganus* develop larger song repertoire than when reared singly as a result of vocal improvization without imitation [57,58]. So the simple fact of being acoustically stimulated by social sounds can influence vocal development. As Bengalese finches have higher pitched vocalizations than zebra finches [38], chicks would produce higher pitched calls in this higher pitched context. Following this latter hypothesis, chicks would modify their call structure by imitation of the external model represented by the vocalizations of the adults. This is the mechanism known for song learning, which involves two phases: the memorization of an adult tutor song during the sensory phase followed by the production of initially immature vocalizations that gradually become similar to the tutor song during the sensorimotor phase [59]. In the zebra finch, these two phases happen in succession from day 15 to day 90, with some overlap between day 25 and 60. Our recordings took place from day 5 to day 14, before the onset of the sensorimotor period of song learning. It is thus unlikely that young zebra finches changed their begging call structure by imitation in our experiment, but it remains to be tested.

In all these hypotheses (vocal improvization, vocal imitation or vocal adjustment by social shaping), hearing and auditory feedback are paramount for the structuration of begging calls. Previous studies bolster this idea. Liu *et al.* [60] used a deafening method in chipping sparrow (*Spizella passerina*) chicks to investigate the importance of the auditory feedback in begging call structure. Deafened male chicks lost begging call structuration, emphasizing the importance of auditory feedback. The authors concluded that different pathways for begging call production could exist: one hearing dependent, one not hearing dependent. They also proposed that auditory-sensitive vocal variability during food begging calls could be the first step leading to vocal imitation, as deafened females did not lose begging call structuration and male begging calls were more variable, showing more vocal exploration. Our results confirm that begging calls could participate in early sensorimotor exploration, and it is interesting to note that we found a significantly stronger effect of the cross-fostering on male begging calls than on female begging calls.

Vocal plasticity at such an early age can be surprising. In the zebra finch, song control brain nuclei develop from day 9 in both sexes and start to become sexually dimorphic from day 12 [61]. So, the differential changes of male and female begging calls in our experiment, which happened between day 5 and day 14, are unlikely to be related to the nervous control of song control nuclei. But it could be an early dimorphism in the vocal organ. Adult zebra finch males have modified sound generating structures of the syrinx, which are associated with larger muscles contracting at higher rates [12,13,62]. Together with dimorphism in neural control, these are thought to allow the male to generate vocalizations in a larger frequency range. As dimorphism of the vocal organ starts to develop in the first weeks after hatching [63], begging calls could represent a form of vocal experimentation associated to syrinx maturation.

Furthermore, a fully matured vocal control system may not be necessary to express degrees of vocal plasticity and vocal imitation. Indeed, it has been recently described that social influences on vocal production can act during embryonic stages: in a host species of some cuckoos (the superb fairy wren, *Malurus cyaneus*), females call to their eggs at the end of incubation, and upon hatching nestlings produced begging calls with high similarity to their mother’s call that are used as a password in female-offspring recognition [30].

Our results give new insights into early vocal plasticity in begging calls, and in male–female differences in degrees of plasticity. We show evidences of an adjustment of nestling begging call acoustic characteristics in response to experience during parental care in a non-parasitic passerine species. This adds to the complexity of offspring signalling of needs to the parents. It may help in understanding some of the discrepancies in the relationships between begging intensity and parental response [14,15] as well as between begging characteristics and several proxies of nestlings’ needs [12,16–18]. To evaluate the impact of reinforcement learning on parents–offspring conflict, future studies might benefit from analysing parents–offspring interactions as time series or using dynamical systems theory.

## Supplementary Material

Parental Influence on Begging Calls - ESM

## Data accessibility

Data available from Dryad at http://dx.doi.org/10.5061/dryad.9pn24.
