# Supplementary material for: Parental influence on begging call structure in zebra finches (Taeniopygia guttata): evidence of early vocal plasticity
Source: R Soc Open Sci. 2015 Nov 25;2(11):150497. doi: 10.1098/rsos.150497 (PMC4680624; doi:10.1098/rsos.150497)

Electronic Supplementary Material

1. MATERIAL AND METHODS

Table ESM1: Composition of experimental groups, ZFR = chicks reared by zebra finch parents and BFR = chicks reared by Bengalese finch parents. Number of subjects of each sex recorded at each age (F= Females, M= Males).

| **Age** | 5 | 6 | 7 | 8 | 9 |
| --- | --- | --- | --- | --- | --- |
| **ZFR** | 1 F - 0 M | 3 F - 1 M | 2 F - 2 M | 1 F - 2 M | 1 F - 1 M |
| **BFR** | 4 F - 2 M | 1 F - 3 M | 2 F - 1 M | 3 F - 0 M | 4 F - 4 M |
|  |  |  |  |  |  |
| **Age** | 10 | 11 | 12 | 13 | 14 |
| **ZFR** | 5 F - 3 M | 2 F - 1 M | 2 F - 3 M | 2 F - 3 M | 3 F - 2 M |
| **BFR** | 2 F - 2 M | 6 F - 3 M | -- | 2 F - 2 M | 6 F - 4 M |

**Table ESM2: Sex composition of each nest. ZFR = chicks reared by zebra finch parents and BFR = chicks reared by Bengalese finch parents**. The sex ratio was calculated for each brood as the ratio of the number of females over the total number of chicks.

| **Group** | **Foster Nest** | **nF** | **nM** | **Brood Size** | **Intra-Brood Sex-ratio** |
| --- | --- | --- | --- | --- | --- |
| ZFR | 1 | 2 | 2 | 4 | 0.5 |
|  | 3 | 2 | 1 | 3 | 0.67 |
|  | 4 | 2 | 1 | 3 | 0.67 |
|  | 6 | 2 | 1 | 3 | 0.67 |
|  | 7 | 1 | 2 | 3 | 0.33 |
|  | 8 | 1 | 2 | 3 | 0.33 |
|  | 9 | 2 | 1 | 3 | 0.67 |
|  | 10 | 1 | 1 | 2 | 0.5 |
|  | 11 | 2 | 1 | 3 | 0.67 |
|  | 15 | 1 | 1 | 2 | 0.5 |
|  | 16 | 2 | 0 | 2 | 1 |
| BFR | 1 | 1 | 2 | 3 | 0.33 |
|  | 2 | 1 | 2 | 3 | 0.33 |
|  | 3 | 2 | 1 | 3 | 0.67 |
|  | 4 | 1 | 2 | 3 | 0.33 |
|  | 5 | 2 | 1 | 3 | 0.67 |
|  | 6 | 3 | 1 | 4 | 0.75 |
|  | 7 | 1 | 2 | 3 | 0.33 |
|  | 8 | 2 | 1 | 3 | 0.67 |

**Table ESM3: Composition of the begging call dataset. a-ZFR = chicks reared by zebra finch parents and b-BFR = chicks reared by Bengalese finch parents. NCalls = number of calls per session for each chick. Age in days post-hatching.**

| **a-** | **ZFR Group** | | | | **b-** | **BFR Group** | | | |
| --- | --- | --- | --- | --- | --- | --- | --- | --- | --- |
|  | **Subject** | **Sex** | **Age** | **Ncalls** |  | **Subject** | **Sex** | **Age** | **Ncalls** |
|  | 318 | M | 12 | 38 |  | 427 | M | 10 | 14 |
|  | 319 | F | 11 | 72 |  |  |  | 13 | 111 |
|  | 321 | F | 12 | 10 |  | 429 | F | 8 | 30 |
|  | 323 | M | 12 | 159 |  |  |  | 10 | 74 |
|  |  | M | 13 | 137 |  | 430 | M | 10 | 70 |
|  | 339 | F | 6 | 87 |  |  |  | 14 | 147 |
|  |  |  | 10 | 88 |  | 432 | F | 7 | 62 |
|  | 340 | F | 5 | 46 |  |  |  | 9 | 46 |
|  | 341 | M | 8 | 56 |  |  |  | 13 | 82 |
|  | 342 | M | 9 | 147 |  | 433 | F | 9 | 18 |
|  |  |  | 13 | 105 |  |  |  | 10 | 81 |
|  | 343 | F | 6 | 62 |  | 434 | M | 7 | 29 |
|  | 345 | M | 8 | 108 |  |  |  | 9 | 74 |
|  | 347 | F | 10 | 113 |  |  |  | 13 | 96 |
|  | 348 | F | 7 | 95 |  | 435 | F | 5 | 59 |
|  |  |  | 13 | 170 |  |  |  | 9 | 54 |
|  | 349 | M | 7 | 64 |  |  |  | 14 | 194 |
|  |  |  | 13 | 52 |  | 437 | M | 5 | 44 |
|  | 350 | M | 10 | 79 |  |  |  | 9 | 116 |
|  | 352 | M | 7 | 88 |  | 438 | F | 9 | 41 |
|  |  |  | 14 | 99 |  | 439 | F | 8 | 152 |
|  | 354 | F | 8 | 68 |  |  |  | 11 | 158 |
|  |  |  | 10 | 143 |  | 440 | M | 6 | 204 |
|  |  |  | 12 | 157 |  |  |  | 9 | 83 |
|  | 355 | M | 8 | 57 |  | 441 | F | 8 | 180 |
|  |  |  | 10 | 84 |  |  |  | 13 | 50 |
|  |  |  | 12 | 217 |  | 442 | F | 5 | 53 |
|  | 356 | M | 11 | 126 |  |  |  | 7 | 111 |
|  |  |  | 14 | 61 |  |  |  | 11 | 113 |
|  | 357 | F | 11 | 113 |  |  |  | 14 | 25 |
|  |  |  | 14 | 188 |  | 443 | F | 11 | 59 |
|  | 361 | F | 6 | 98 |  |  |  | 14 | 82 |
|  |  |  | 10 | 109 |  | 444 | M | 9 | 93 |
|  | 363 | F | 10 | 166 |  | 445 | F | 5 | 38 |
|  |  |  | 14 | 186 |  |  |  | 11 | 336 |
|  | 364 | F | 9 | 26 |  |  |  | 14 | 264 |
|  |  |  | 13 | 166 |  | 446 | M | 5 | 148 |
|  | 365 | M | 6 | 93 |  |  |  | 11 | 178 |
|  |  |  | 10 | 173 |  |  |  | 14 | 296 |
|  | 366 | F | 7 | 70 |  | 447 | F | 5 | 77 |
|  |  |  | 14 | 97 |  |  |  | 11 | 146 |
|  |  |  |  |  |  |  |  | 14 | 42 |
|  |  |  |  |  |  | 448 | M | 6 | 29 |
|  |  |  |  |  |  |  |  | 11 | 54 |
|  |  |  |  |  |  |  |  | 14 | 127 |
|  |  |  |  |  |  | 450 | M | 6 | 49 |
|  |  |  |  |  |  |  |  | 11 | 102 |
|  |  |  |  |  |  |  |  | 14 | 148 |
|  |  |  |  |  |  | 451 | F | 6 | 30 |
|  |  |  |  |  |  |  |  | 11 | 105 |
|  |  |  |  |  |  |  |  | 14 | 176 |

**2. RESULTS**

**Table ESM4: Statistical table of models on principal components of the PCA on acoustic parameters.** Estimates, standard errors and confidence intervals, generated with ‘lsmeans’ function (‘lmerTest” R package) for each model. a-Values resulting from the initial full model, composed of three fixed factors (Group, Age and Sex). b- When the triple interaction was significant, post hoc models were run in subsets of the data with two fixed factors (in males and females separately to test for the Group: Age interaction, and in young and old nestlings separately to test for the Group: Sex interaction).

| **a- INITIAL FULL MODEL : Group*Age*Sex** | | | | | |  |
| --- | --- | --- | --- | --- | --- | --- |
|  | **Estimate** | **Standard Error** | **DF** | **Lower CI** | **Upper CI** |  |
| *PC1- all data* |  |  |  |  |  |  |
| ZFR group | 0.925 | 0.260 | 16.400 | 0.376 | 1.474 |  |
| BFR group | -0.862 | 0.277 | 9.800 | -1.480 | -0.244 |  |
| Females | 0.304 | 0.289 | 14.100 | -0.315 | 0.923 |  |
| Males | -0.241 | 0.211 | 11.700 | -0.701 | 0.220 |  |
| ZFR group: Females | 1.074 | 0.396 | 17.800 | 0.241 | 1.906 |  |
| BFR group: Females | -0.466 | 0.422 | 11.800 | -1.387 | 0.456 |  |
| ZFR group: Males | 0.777 | 0.286 | 13.700 | 0.162 | 1.391 |  |
| BFR group: Males | -1.258 | 0.296 | 8.000 | -1.942 | -0.574 |  |
|  |  |  |  |  |  |  |
| *PC2- all data* |  |  |  |  |  |  |
| ZFR Group | 0.5601 | 0.2498 | 12.1 | 0.0163 | 1.1039 |  |
| BFR Group | -0.2665 | 0.2903 | 8.6 | -0.9274 | 0.3944 |  |
| Females | 0.4696 | 0.2209 | 22 | 0.0114 | 0.9277 |  |
| Males | -0.176 | 0.2408 | 27.9 | -0.6693 | 0.3173 |  |
| ZFR Group: Females | 0.7618 | 0.3032 | 20 | 0.1292 | 1.3944 |  |
| BFR Group: Males | 0.1773 | 0.3201 | 11.9 | -0.5204 | 0.8751 |  |
| ZFR Group: Males | 0.3584 | 0.3155 | 21.6 | -0.2966 | 1.0134 |  |
| BFR Group: Males | -0.7103 | 0.3528 | 17.5 | -1.453 | 0.0324 |  |
|  |  |  |  |  |  |  |
| **b- POST HOC MODELS FOLLOWING SIGNIFICANT GROUP*AGE*SEX INTERACTION** | | | | | | |
|  | **Estimate** | **Standard Error** | **DF** | **Lower CI** | **Upper CI** |  |
| *PC1- post hoc test on males* |  |  |  |  |  |  |
| ZFR group | 0.846 | 0.258 | 16.900 | 0.301 | 1.390 |  |
| BFR group | -1.249 | 0.265 | 12.900 | -1.822 | -0.676 |  |
|  |  |  |  |  |  |  |
| *PC1- post hoc test on females* |  |  |  |  |  |  |
| ZFR group | 1.010 | 0.397 | 17.300 | 0.174 | 1.845 |  |
| BFR group | -0.476 | 0.410 | 8.500 | -1.412 | 0.460 |  |
|  |  |  |  |  |  |  |
| *PC1- post hoc test on young nestlings* |  |  |  |  |  |  |
| ZFR group | -0.768 | 0.519 | 8.100 | -1.963 | 0.428 |  |
| BFR group | -2.182 | 0.497 | 6.200 | -3.391 | -0.973 |  |
| Females | -1.007 | 0.397 | 9.100 | -1.903 | -0.111 |  |
| Males | -1.942 | 0.444 | 13.900 | -2.896 | -0.989 |  |
| ZFR group: Females | -0.184 | 0.560 | 9.900 | -1.433 | 1.066 |  |
| BFR group: Females | -1.831 | 0.558 | 8.700 | -3.100 | -0.563 |  |
| ZFR group: Males | -1.352 | 0.688 | 15.100 | -2.816 | 0.113 |  |
| BFR group: Males | -2.533 | 0.555 | 10.400 | -3.763 | -1.303 |  |
|  |  |  |  |  |  |  |
| *PC1- post hoc test on old nestlings* |  |  |  |  |  |  |
| ZFR group | 2.042 | 0.350 | 12.200 | 1.280 | 2.805 |  |
| BFR group | 0.667 | 0.399 | 5.200 | -0.345 | 1.680 |  |
| Females | 1.372 | 0.397 | 4.100 | 0.282 | 2.461 |  |
| Males | 1.338 | 0.360 | 11.200 | 0.548 | 2.128 |  |
| ZFR group: Females | 1.340 | 0.515 | 14.800 | 0.241 | 2.439 |  |
| BFR group: Females | 1.404 | 0.596 | 1.600 | -1.789 | 4.597 |  |
| ZFR group: Males | 2.745 | 0.470 | 11.200 | 1.714 | 3.776 |  |
| BFR group: Males | -0.069 | 0.548 | 11.400 | -1.271 | 1.133 |  |

**Figure ESM1: Individual developmental trajectories of begging call acoustic features in males and females.** Each point represents the individual mean of the first principal component (PC1) calculated from all the calls recorded for this individual at this age. The error bars are standard error of the mean (related to the number of calls analysed). Each point has error bars, usually very small illustrating the stability of begging call structure for an individual at a given age stage. Lines connect points at different ages of the same individual. Linear mixed effect models were performed on PC1. Because triple interaction of sex, group and age was significant, post hoc models were then performed separating males and females (see table ESM4). The models showed differential developmental trajectories (Age: Group interaction) between cross-fostering groups in males but not in females. The significance of Age:Group interaction is indicated in insert. *: P≤0.05.


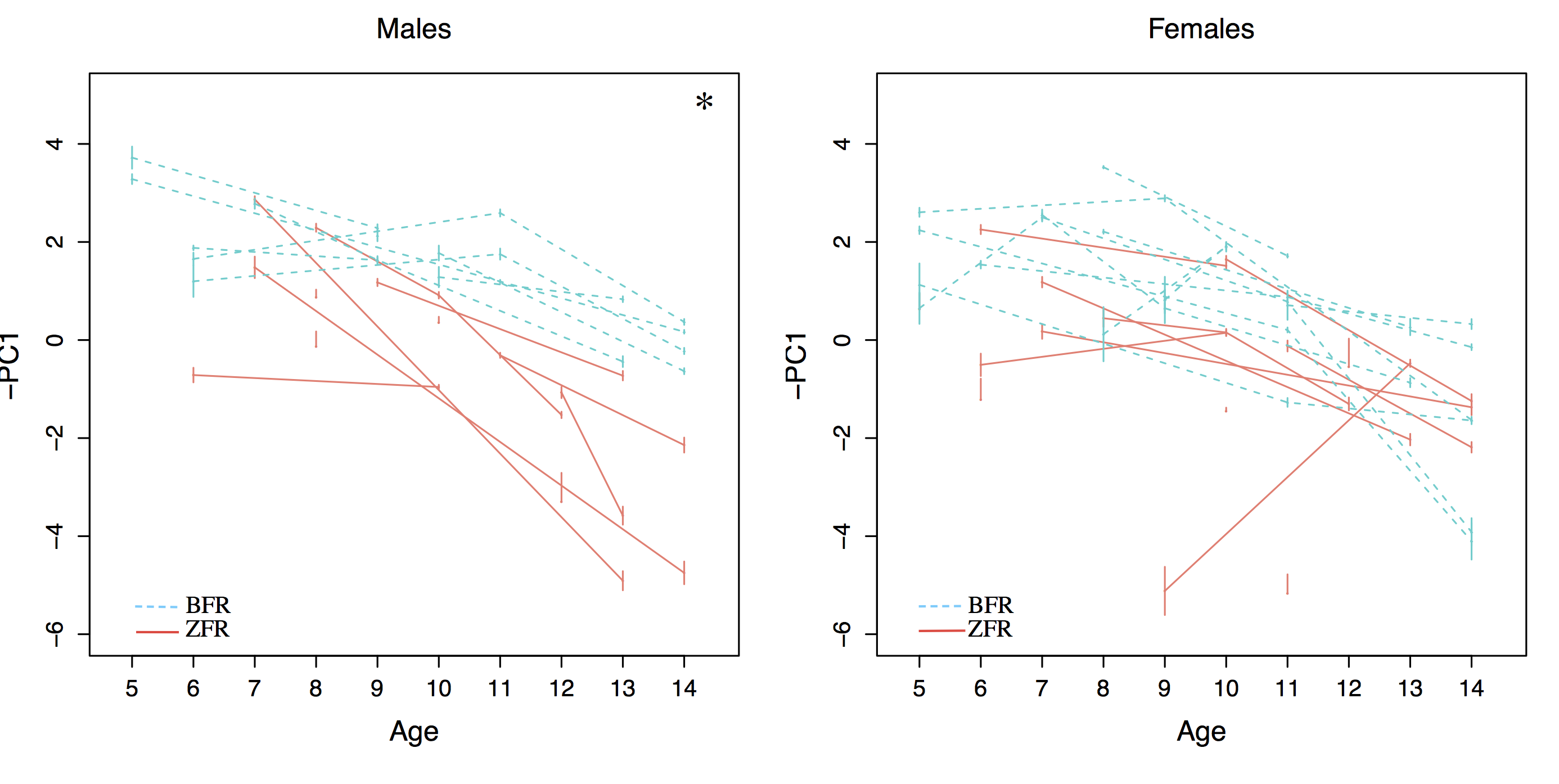


**Figure ESM 2: Effect of call index during recording session on principal components of the PCA on acoustic parameters**. Statistics showed an effect of the call index on PC2 (table ESM4) but also on PC1 in males only. Call index was centered and reduced. Since no effect of the cross-fostering group was found on PC2, all data are pooled together. On the contrary, because PC1 was significantly affected by the cross-fostering group in males, groups are plotted separately to check possible interactions. The significance of the effect of the call index on PC values is indicated in insert. ***: P≤0.001, *: P≤0.05, . : P P≤0.1.

**
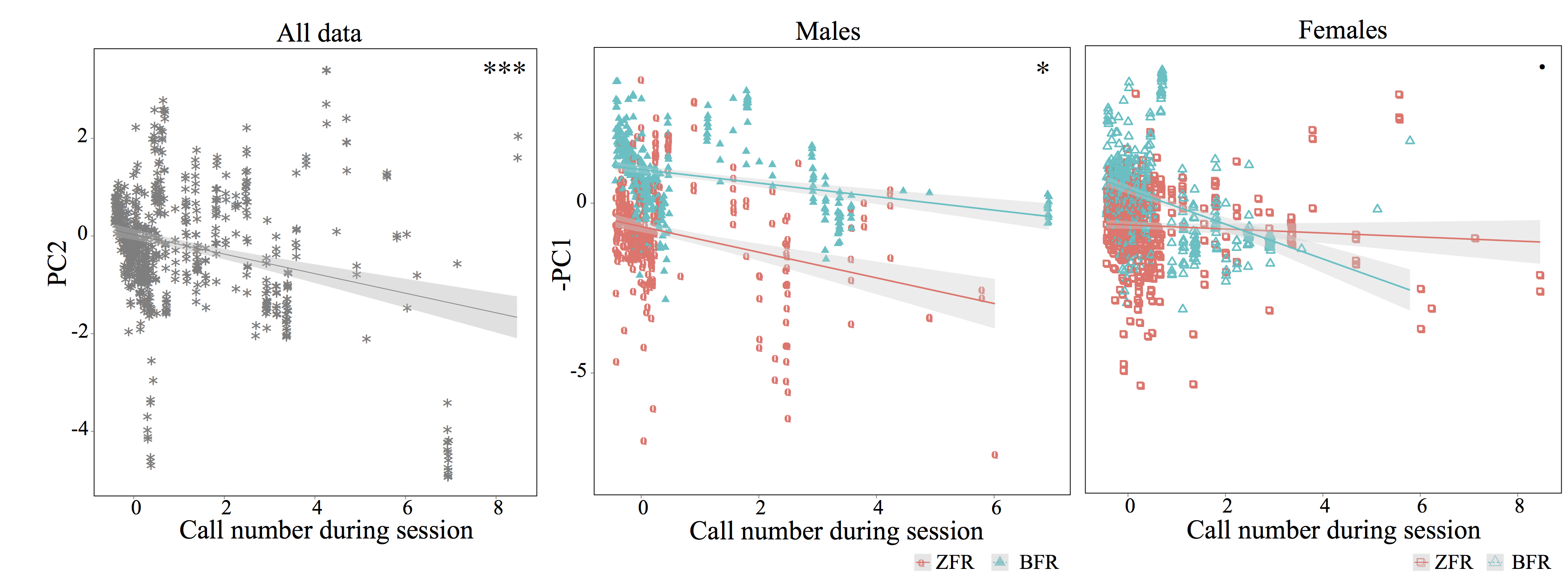
**

**Figure ESM3: Examples of begging call spectro-temporal features of zebra finch chicks either fostered to zebra finch parents (ZFR) or Bengalese finch parents (BFR).** Spectra (left column) and spectrograms (right colum) of two females at day 5 (F1 and F2) and two males at day 8/9 (M1 and M1). Note the decrease in call frequency bandwidth between 5 and 8/9 DPH.


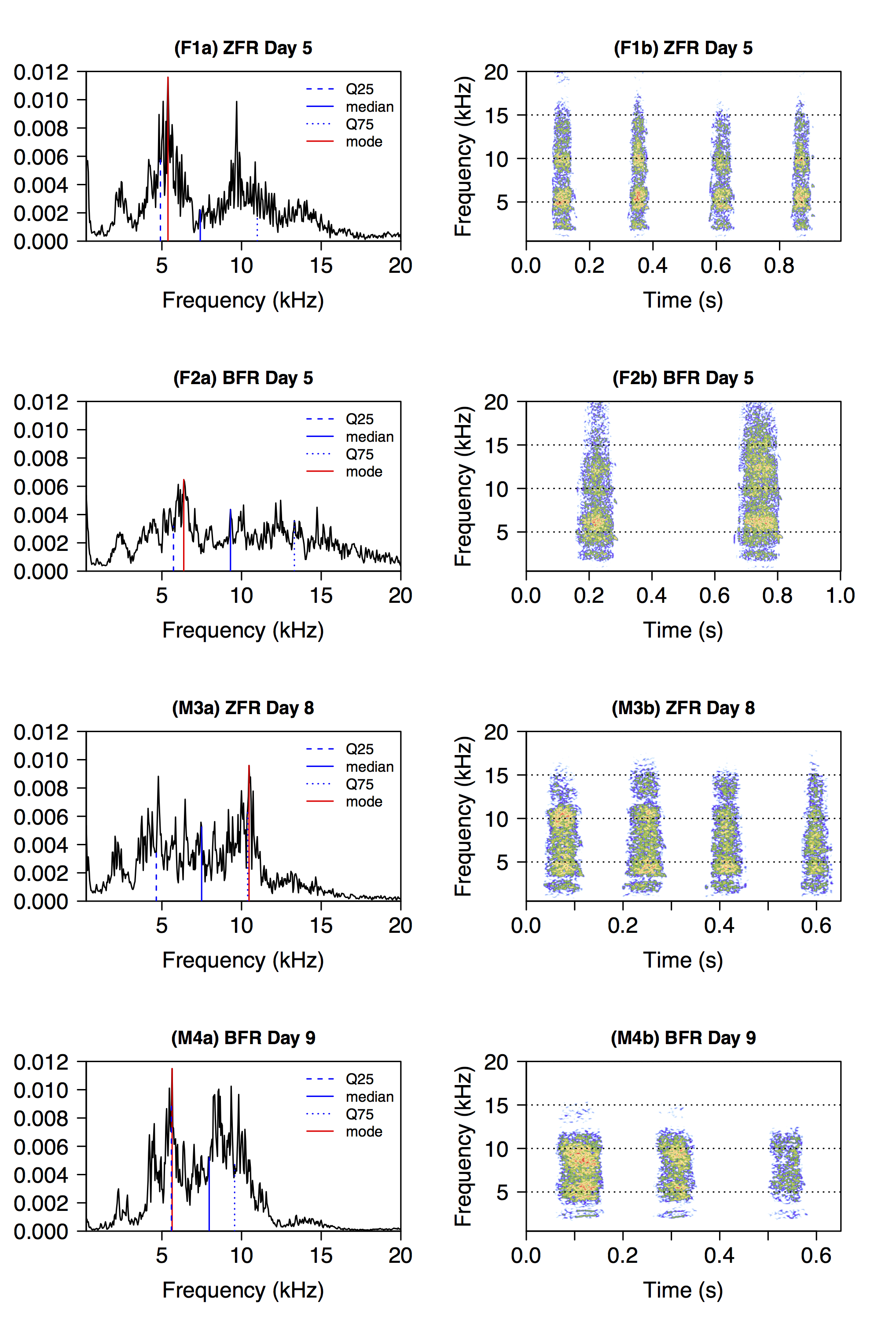


Table ESM5: Acoustic features of begging calls of chicks reared by zebra finch parents (=ZFR) or chicks reared by Bengalese finch parents (=BFR), recorded from 6 to 14 DPH. Values are means of the mean values obtained per individual ± Se, standard error calculated using the number of subjects in the subset of data, a NA as standard error indicates that only one individual composed the subset. The number of individuals included per age, sex and group is specified in table 1. Only age classes presenting a complete comparison (both sexes and both cross-fostering groups) are presented.

| **Age** | **Group** | **Sex** | **Mean^1^** | **Se** | **Sd^1^** | **Se** | **Median^1^** | **Se** | **Mode^1^** | **Se** | **Q25^1^** | **Se** | **Q75^1^** | **Se** | **IQR^1^** | **Se** | **Skewness** | **Se** | **Sfm** | **Se** | **Duration^2^** | **Se** |
| --- | --- | --- | --- | --- | --- | --- | --- | --- | --- | --- | --- | --- | --- | --- | --- | --- | --- | --- | --- | --- | --- | --- |
| 6 | ZFR | F | 6.83 | 0.58 | 3.50 | 0.01 | 6.28 | 0.61 | 4.70 | 0.59 | 4.19 | 0.58 | 9.46 | 0.80 | 5.27 | 0.23 | 1.79 | 0.25 | 0.72 | 0.02 | 58.41 | 11.93 |
|  |  | M | 6.51 | NA | 3.24 | NA | 5.94 | NA | 4.08 | NA | 4.07 | NA | 8.57 | NA | 4.50 | NA | 2.18 | NA | 0.72 | NA | 44.07 | NA |
|  | BFR | F | 7.41 | NA | 3.52 | NA | 6.58 | NA | 5.32 | NA | 4.72 | NA | 10.17 | NA | 5.45 | NA | 1.05 | NA | 0.80 | NA | 96.24 | NA |
|  |  | M | 7.50 | 0.12 | 3.62 | 0.02 | 7.04 | 0.18 | 4.97 | 0.31 | 4.59 | 0.08 | 10.50 | 0.15 | 5.91 | 0.17 | 1.47 | 0.20 | 0.80 | 0.02 | 83.38 | 6.41 |
| 7 | ZFR | F | 6.98 | 0.23 | 3.37 | 0.08 | 6.57 | 0.37 | 5.46 | 0.88 | 4.42 | 0.26 | 9.37 | 0.24 | 4.96 | 0.03 | 1.10 | 0.25 | 0.77 | 0.01 | 52.38 | 1.08 |
|  |  | M | 7.98 | 0.48 | 3.44 | 0.08 | 7.67 | 0.54 | 5.85 | 0.00 | 5.33 | 0.44 | 10.73 | 0.63 | 5.40 | 0.19 | 1.17 | 0.07 | 0.75 | 0.03 | 74.01 | 21.58 |
|  | BFR | F | 7.98 | 0.01 | 3.37 | 0.11 | 7.97 | 0.02 | 7.55 | 0.72 | 5.26 | 0.10 | 10.68 | 0.01 | 5.41 | 0.10 | 0.86 | 0.06 | 0.75 | 0.04 | 69.64 | 0.53 |
|  |  | M | 8.35 | NA | 3.43 | NA | 8.54 | NA | 6.56 | NA | 5.49 | NA | 11.18 | NA | 5.69 | NA | 1.04 | NA | 0.74 | NA | 58.05 | NA |
| 10 | ZFR | F | 6.85 | 0.31 | 3.12 | 0.12 | 6.67 | 0.41 | 5.86 | 0.78 | 4.44 | 0.35 | 8.98 | 0.36 | 4.53 | 0.26 | 1.08 | 0.10 | 0.70 | 0.03 | 52.96 | 5.80 |
|  |  | M | 6.71 | 0.31 | 2.91 | 0.18 | 6.53 | 0.42 | 6.27 | 0.73 | 4.62 | 0.26 | 8.60 | 0.45 | 3.98 | 0.31 | 1.34 | 0.21 | 0.63 | 0.05 | 65.05 | 10.11 |
|  | BFR | F | 7.62 | 0.07 | 3.33 | 0.17 | 7.62 | 0.21 | 7.13 | 0.17 | 5.05 | 0.41 | 10.20 | 0.06 | 5.15 | 0.47 | 0.91 | 0.02 | 0.75 | 0.05 | 56.64 | 1.64 |
|  |  | M | 7.52 | 0.18 | 3.28 | 0.31 | 7.62 | 0.22 | 6.51 | 0.16 | 5.00 | 0.22 | 9.88 | 0.60 | 4.88 | 0.82 | 1.14 | 0.02 | 0.73 | 0.03 | 57.86 | 19.26 |
| 11 | ZFR | F | 5.27 | 1.16 | 3.25 | 0.15 | 4.65 | 1.55 | 3.90 | 2.24 | 2.88 | 1.19 | 7.24 | 1.45 | 4.36 | 0.26 | 2.62 | 1.58 | 0.58 | 0.11 | 42.27 | 0.30 |
|  |  | M | 6.44 | NA | 3.10 | NA | 6.17 | NA | 4.58 | NA | 4.04 | NA | 8.71 | NA | 4.67 | NA | 1.15 | NA | 0.67 | NA | 52.28 | NA |
|  | BFR | F | 6.97 | 0.27 | 3.10 | 0.16 | 6.75 | 0.37 | 5.99 | 0.59 | 4.62 | 0.35 | 9.05 | 0.31 | 4.44 | 0.36 | 1.28 | 0.06 | 0.68 | 0.04 | 62.27 | 7.88 |
|  |  | M | 7.72 | 0.17 | 3.04 | 0.35 | 7.75 | 0.13 | 7.11 | 0.51 | 5.49 | 0.37 | 9.92 | 0.63 | 4.43 | 0.92 | 1.02 | 0.21 | 0.66 | 0.09 | 77.37 | 16.28 |
| 13 | ZFR | F | 5.92 | 0.51 | 3.12 | 0.46 | 5.52 | 0.90 | 3.94 | 1.75 | 3.48 | 1.14 | 8.27 | 0.05 | 4.80 | 1.19 | 1.71 | 0.25 | 0.59 | 0.08 | 70.05 | 8.44 |
|  |  | M | 5.20 | 0.63 | 3.15 | 0.26 | 4.62 | 0.99 | 3.43 | 1.53 | 2.85 | 0.90 | 7.16 | 0.56 | 4.32 | 0.64 | 3.24 | 0.86 | 0.52 | 0.03 | 45.96 | 4.08 |
|  | BFR | F | 6.34 | 0.14 | 2.98 | 0.31 | 6.19 | 0.03 | 5.71 | 0.28 | 4.23 | 0.19 | 8.19 | 0.70 | 3.95 | 0.89 | 1.33 | 0.38 | 0.66 | 0.08 | 70.85 | 0.79 |
|  |  | M | 6.68 | 0.51 | 2.89 | 0.11 | 6.57 | 0.50 | 5.91 | 0.73 | 4.69 | 0.90 | 8.57 | 0.17 | 3.88 | 0.73 | 1.03 | 0.17 | 0.63 | 0.01 | 72.32 | 10.99 |
| 14 | ZFR | F | 5.68 | 0.15 | 3.06 | 0.26 | 5.38 | 0.17 | 4.08 | 0.83 | 3.33 | 0.24 | 7.93 | 0.51 | 4.61 | 0.72 | 1.50 | 0.21 | 0.59 | 0.05 | 49.53 | 2.73 |
|  |  | M | 4.91 | 0.65 | 3.41 | 0.44 | 3.98 | 1.16 | 2.54 | 1.27 | 2.36 | 1.08 | 6.89 | 0.55 | 4.53 | 0.53 | 3.16 | 0.74 | 0.58 | 0.02 | 55.52 | 7.34 |
|  | BFR | F | 5.71 | 0.35 | 3.18 | 0.08 | 5.16 | 0.47 | 3.81 | 0.57 | 3.32 | 0.40 | 7.83 | 0.47 | 4.52 | 0.20 | 2.16 | 0.56 | 0.61 | 0.04 | 50.29 | 7.29 |
|  |  | M | 6.57 | 0.20 | 2.81 | 0.11 | 6.35 | 0.20 | 5.93 | 0.52 | 4.53 | 0.36 | 8.44 | 0.18 | 3.92 | 0.41 | 1.23 | 0.31 | 0.59 | 0.02 | 79.77 | 14.41 |

**^1^=** in kHz, **^2^** = in ms

**Table ESM6: Statistical results from models on each acoustic parameter separately. The table depicts values resulting from the ‘drop1’ function (‘lmerTest’ R package) computed on the full model testing the following interaction explanatory variables: Group*Age*Sex. ‘Drop1” computes all the single terms that can be added to or dropped from the model, fits those models and computes a table of the changes in fit. Only relevant interactions then compose the table. When a significant triple interaction was found, post hoc tests were run first in the two sexes (Test of the group : Age interaction, table …) and on two age classes (test of the group : Sex interaction, table…).**

|  | **DF** | **LRT** | **Pr(Chi)** |  |  | **DF** | **LRT** | **Pr(Chi)** |
| --- | --- | --- | --- | --- | --- | --- | --- | --- |
|  |  |  |  |  |  |  |  |  |
| *Mean frequency* |  |  |  |  | *Third Quartile frequency (Q75)* |  |  |  |
| Call number | 1 | 1.193 | 0.275 |  | Call number | 1 | 3.622 | 0.057 |
| Intra-brood sex-ratio | 1 | 0.290 | 0.591 |  | Intra-brood sex-ratio | 1 | 0.048 | 0.826 |
| BCI | 1 | 0.047 | 0.828 |  | BCI | 1 | 0.894 | 0.344 |
| Social Group: Age: Sex | 1 | 9.707 | **0.002** |  | Social Group: Age: Sex | 1 | 8.151 | **0.004** |
|  |  |  |  |  |  |  |  |  |
| *Standard deviation (Sd)* | |  |  |  | *Inter-quartile-Range (IQR)* |  |  |  |
| Call number | 1 | 66.385 | **0.000** |  | Call number | 1 | 50.015 | **0.000** |
| Intra-brood sex-ratio | 1 | 3.035 | 0.081 |  | Intra-brood sex-ratio | 1 | 0.888 | 0.346 |
| BCI | 1 | 0.095 | 0.758 |  | BCI | 1 | 0.001 | 0.975 |
| Social Group: Age: Sex | 1 | 4.086 | **0.043** |  | Social Group: Age: Sex | 1 | 0.056 | 0.812 |
|  |  |  |  |  |  |  |  |  |
| *Median frequency* |  |  |  |  | *Spectral Skewness* |  |  |  |
| Call number | 1 | 1.853 | 0.173 |  | Call number | 1 | 1.928 | 0.165 |
| Intra-brood sex-ratio | 1 | 1.529 | 0.216 |  | Intra-brood sex-ratio | 1 | 1.909 | 0.167 |
| BCI | 1 | 0.047 | 0.828 |  | BCI | 1 | 1.748 | 0.186 |
| Social Group: Age: Sex | 1 | 8.646 | **0.003** |  | Social Group: Age: Sex | 1 | 8.019 | **0.005** |
|  |  |  |  |  |  |  |  |  |
| *Mode frequency* |  |  |  |  | *Spectral flatness* |  |  |  |
| Call number | 1 | 8.053 | **0.005** |  | Call number | 1 | 23.084 | **0.000** |
| Intra-brood sex-ratio | 1 | 3.468 | 0.063 |  | Intra-brood sex-ratio | 1 | 0.663 | 0.416 |
| BCI | 1 | 0.255 | 0.613 |  | BCI | 1 | 0.014 | 0.904 |
| Social Group: Age: Sex | 1 | 6.665 | **0.010** |  | Social Group: Age: Sex | 1 | 0.063 | 0.801 |
|  |  |  |  |  |  |  |  |  |
| *First Quartile frequency (Q25)* | | |  |  | *Call duration* |  |  |  |
| Call number | 1 | 33.912 | **0.000** |  | Call number | 1 | 2.098 | 0.147 |
| Intra-brood sex-ratio | 1 | 1.678 | 0.195 |  | Intra-brood sex-ratio | 1 | 1.008 | 0.315 |
| BCI | 1 | 0.320 | 0.571 |  | BCI | 1 | 1.205 | 0.272 |
| Social Group: Age: Sex | 1 | 8.606 | **0.003** |  | Social Group: Age: Sex | 1 | 5.145 | **0.023** |

**Table ESM7: Statistical results of models on each acoustic parameter, within sexes post hoc models.**

|  | **MALES** | | |  | **FEMALES** | | |  |
| --- | --- | --- | --- | --- | --- | --- | --- | --- |
|  | **DF** | **LRT** | **Pr(Chi)** |  | **DF** | **LRT** | **Pr(Chi)** |  |
|  |  |  |  |  |  |  |  |  |
| *Mean frequency* |  |  |  |  |  |  |  |  |
| Call number | 1 | 1.030 | 0.310 |  | 1 | 3.072 | 0.080 |  |
| Intra-brood sex-ratio | 1 | -0.048 | 1.000 |  | 1 | 0.704 | 0.402 |  |
| BCI | 1 | 0.004 | 0.950 |  | 1 | 0.011 | 0.915 |  |
| Social Group: Age | 1 | 3.165 | 0.075 |  | 1 | 1.072 | 0.301 |  |
|  |  |  |  |  |  |  |  |  |
| *Standard deviation (Sd)* | |  |  |  |  |  |  |  |
| Call number | 1 | 130.367 | **0.000** |  | 1 | 6.063 | **0.014** |  |
| Intra-brood sex-ratio | 1 | 0.826 | 0.364 |  | 1 | 3.816 | 0.051 |  |
| BCI | 1 | 0.047 | 0.828 |  | 1 | 0.410 | 0.522 |  |
| Social Group: Age | 1 | 6.304 | **0.012** |  | 1 | 0.027 | 0.870 |  |
|  |  |  |  |  |  |  |  |  |
| *Median frequency* |  |  |  |  |  |  |  |  |
| Call number | 1 | 0.274 | 0.600 |  | 1 | 3.181 | 0.074 |  |
| Intra-brood sex-ratio | 1 | 0.225 | 0.636 |  | 1 | 1.261 | 0.261 |  |
| BCI | 1 | 0.392 | 0.531 |  | 1 | 0.458 | 0.498 |  |
| Social Group: Age | 1 | 4.512 | **0.034** |  | 1 | 0.885 | 0.347 |  |
|  |  |  |  |  |  |  |  |  |
| *Mode frequency* |  |  |  |  |  |  |  |  |
| Call number | 1 | 2.135 | 0.144 |  | 1 | 5.861 | 0.015 |  |
| Intra-brood sex-ratio | 1 | 0.095 | 0.758 |  | 1 | 4.149 | 0.042 |  |
| BCI | 1 | 0.338 | 0.561 |  | 1 | 0.770 | 0.380 |  |
| Social Group: Age | 1 | 6.577 | **0.010** |  | 1 | 0.564 | 0.453 |  |
|  |  |  |  |  |  |  |  |  |
| *First Quartile frequency (Q25)* | | |  |  |  |  |  |  |
| Call number | 1 | 32.334 | **0.000** |  | 1 | 11.609 | **0.001** |  |
| Intra-brood sex-ratio | 1 | 0.219 | 0.640 |  | 1 | 2.237 | 0.135 |  |
| BCI | 1 | 0.222 | 0.637 |  | 1 | 0.078 | 0.779 |  |
| Social Group: Age | 1 | 6.199 | **0.013** |  | 1 | 0.334 | 0.563 |  |
|  |  |  |  |  |  |  |  |  |
| *Third Quartile frequency (Q75)* | | |  |  |  |  |  |  |
| Call number | 1 | 16.581 | **0.000** |  | 1 | 0.028 | 0.868 |  |
| Intra-brood sex-ratio | 1 | 0.946 | 0.331 |  | 1 | 0.127 | 0.722 |  |
| BCI | 1 | 0.171 | 0.679 |  | 1 | 0.031 | 0.861 |  |
| Social Group: Age | 1 | 0.759 | 0.384 |  | 1 | 3.754 | 0.053 |  |
|  |  |  |  |  |  |  |  |  |
| *Spectral Skewness* |  |  |  |  |  |  |  |  |
| Call number | 1 | 2.439 | 0.118 |  | 1 | 6.411 | **0.011** |  |
| Intra-brood sex-ratio | 1 | 0.608 | 0.435 |  | 1 | 1.545 | 0.214 |  |
| BCI | 1 | 0.426 | 0.514 |  | 1 | 2.013 | 0.156 |  |
| Social Group: Age | 1 | 6.217 | **0.013** |  | 1 | 1.183 | 0.277 |  |
|  |  |  |  |  |  |  |  |  |
| *Call duration* |  |  |  |  |  |  |  |  |
| Call number | 1 | 3.540 | 0.060 |  | 1 | 0.249 | 0.618 |  |
| Intra-brood sex-ratio | 1 | 0.157 | 0.692 |  | 1 | 5.731 | **0.017** |  |
| BCI | 1 | 5.171 | **0.023** |  | 1 | 0.931 | 0.335 |  |
| Social Group: Age | 1 | 0.269 | 0.604 |  | 1 | 4.280 | **0.039** |  |

**Table ESM8: Statistical results from models on each acoustic parameter, post hoc models separating age classes (young nestlings : 5≤Age≤7, old nestlings : 12≤Age≤15).** When the two way interaction between Group and Sex was significant, which was the case in old neslting for seven of the eight parameters (a) post hoc test were run separating sexes (b).

| **a- POST HOC MODELS SEPARATING YOUNG AND OLD NESTLINGS** | | | | | | | |  | **b- POST HOC MODELS IN OLD NESTLING** | | | | | | | |
| --- | --- | --- | --- | --- | --- | --- | --- | --- | --- | --- | --- | --- | --- | --- | --- | --- |
|  | **YOUNG** | | |  | **OLD** | | |  |  | **MALE OLD NESTLINGS** | | |  | **FEMALE OLD NESTLINGS** | | |
|  | **DF** | **LRT** | **Pr(Chi)** |  | **DF** | **LRT** | **Pr(Chi)** |  |  | **Df** | **LRT** | **Pr(Chi)** |  | **Df** | **LRT** | **Pr(Chi)** |
|  |  |  |  |  |  |  |  |  |  |  |  |  |  |  |  |  |
| *Mean frequency* |  |  |  |  |  |  |  |  | *Mean frequency* | |  |  |  |  |  |  |
| Call number | 1 | 0.016 | 0.898 |  | 1 | 0.443 | 0.506 |  | Social Group | 1 | 8.286 | **0.004** |  | 1 | 0.001 | 0.969 |
| Intra-brood sex-ratio | 1 | 0.431 | 0.512 |  | 1 | 0.014 | 0.907 |  | Call number | 1 | 0.587 | 0.443 |  | 1 | 4.496 | **0.034** |
| BCI | 1 | 0.406 | 0.524 |  | 1 | 0.054 | 0.817 |  | Intra-brood sex-ratio | 1 | 0.651 | 0.420 |  | 1 | 0.249 | 0.618 |
| Social Group: Sex | 1 | 0.728 | 0.393 |  | 1 | 6.966 | **0.008** |  | BCI | 1 | 0.409 | 0.523 |  | 1 | 0.028 | 0.868 |
|  |  |  |  |  |  |  |  |  |  |  |  |  |  |  |  |  |
| *Standard deviation (Sd)* | |  |  |  |  |  |  |  |  |  |  |  |  |  |  |  |
| Call number | 1 | 0.292 | 0.589 |  | 1 | 86.962 | **0.000** |  |  |  |  |  |  |  |  |  |
| Intra-brood sex-ratio | 1 | 1.023 | 0.312 |  | 1 | 1.048 | 0.306 |  |  |  |  |  |  |  |  |  |
| BCI | 1 | 2.128 | 0.145 |  | 1 | 0.020 | 0.888 |  |  |  |  |  |  |  |  |  |
| Social Group: Sex | 1 | 1.949 | 0.163 |  | 1 | 1.757 | 0.185 |  |  |  |  |  |  |  |  |  |
|  |  |  |  |  |  |  |  |  |  |  |  |  |  |  |  |  |
| *Median frequency* |  |  |  |  |  |  |  |  | *Median frequency* | |  |  |  |  |  |  |
| Call number | 1 | 0.303 | 0.582 |  | 1 | 0.833 | 0.361 |  | Social Group | 1 | 8.163 | **0.004** |  | 1 | 0.010 | 0.921 |
| Intra-brood sex-ratio | 1 | 0.405 | 0.524 |  | 1 | 0.201 | 0.654 |  | Call number | 1 | 0.009 | 0.926 |  | 1 | 2.723 | 0.099 |
| BCI | 1 | 2.239 | 0.135 |  | 1 | 0.054 | 0.816 |  | Intra-brood sex-ratio | 1 | 0.804 | 0.370 |  | 1 | 0.106 | 0.745 |
| Social Group: Sex | 1 | 0.117 | 0.732 |  | 1 | 6.831 | **0.009** |  | BCI | 1 | 0.151 | 0.697 |  | 1 | 0.138 | 0.711 |
|  |  |  |  |  |  |  |  |  |  |  |  |  |  |  |  |  |
| *Mode frequency* |  |  |  |  |  |  |  |  | *Mode frequency* | |  |  |  |  |  |  |
| Call number | 1 | 0.002 | 0.968 |  | 1 | 13.361 | **0.000** |  | Social Group | 1 | 11.396 | **0.001** |  | 1 | 0.519 | 0.471 |
| Intra-brood sex-ratio | 1 | 0.053 | 0.818 |  | 1 | 3.251 | 0.071 |  | Call number | 1 | 6.019 | **0.014** |  | 1 | 8.968 | **0.003** |
| BCI | 1 | 0.862 | 0.353 |  | 1 | 1.860 | 0.173 |  | Intra-brood sex-ratio | 1 | 0.082 | 0.775 |  | 1 | 0.567 | 0.451 |
| Social Group: Sex | 1 | 0.094 | 0.759 |  | 1 | 4.843 | **0.028** |  | BCI | 1 | 0.407 | 0.524 |  | 1 | 0.265 | 0.607 |
|  |  |  |  |  |  |  |  |  |  |  |  |  |  |  |  |  |
| *First Quartile frequency (Q25)* | | |  |  |  |  |  |  | *First Quartile frequency (Q25)* | | | |  |  |  |  |
| Call number | 1 | 0.004 | 0.952 |  | 1 | 42.136 | **0.000** |  | Social Group | 1 | 8.174 | **0.004** |  | 1 | 0.494 | 0.482 |
| Intra-brood sex-ratio | 1 | 0.129 | 0.719 |  | 1 | 0.242 | 0.623 |  | Call number | 1 | 31.137 | **0.000** |  | 1 | 17.017 | **0.000** |
| BCI | 1 | 0.296 | 0.587 |  | 1 | 0.149 | 0.699 |  | Intra-brood sex-ratio | 1 | 0.177 | 0.674 |  | 1 | 0.000 | 0.993 |
| Social Group: Sex | 1 | 1.013 | 0.314 |  | 1 | 4.551 | **0.033** |  | BCI | 1 | 1.242 | 0.265 |  | 1 | 0.096 | 0.757 |
|  |  |  |  |  |  |  |  |  |  |  |  |  |  |  |  |  |
| *Third Quartile frequency (Q75)* | | |  |  |  |  |  |  | *Third Quartile frequency (Q75)* | | | |  |  |  |  |
| Call number | 1 | 0.000 | 0.988 |  | 1 | 5.626 | **0.018** |  | Social Group | 1 | 6.400 | **0.011** |  | 1 | 0.870 | 0.351 |
| Intra-brood sex-ratio | 1 | 0.822 | 0.365 |  | 1 | 0.060 | 0.807 |  | Call number | 1 | 10.041 | **0.002** |  | 1 | 0.234 | 0.629 |
| BCI | 1 | 0.010 | 0.920 |  | 1 | 0.057 | 0.811 |  | Intra-brood sex-ratio | 1 | 0.737 | 0.391 |  | 1 | 0.802 | 0.370 |
| Social Group: Sex | 1 | 2.785 | 0.095 |  | 1 | 5.958 | **0.015** |  | BCI | 1 | 0.131 | 0.717 |  | 1 | 0.578 | 0.447 |
|  |  |  |  |  |  |  |  |  |  |  |  |  |  |  |  |  |
| *Spectral Skewness* |  |  |  |  |  |  |  |  | *Spectral Skewness* | |  |  |  |  |  |  |
| Call number | 1 | 0.072 | 0.788 |  | 1 | 0.019 | 0.890 |  | Social Group | 1 | 12.093 | **0.001** |  | 1 | 0.275 | 0.600 |
| Intra-brood sex-ratio | 1 | 0.068 | 0.794 |  | 1 | 0.183 | 0.669 |  | Call number | 1 | 1.962 | 0.161 |  | 1 | 5.016 | **0.025** |
| BCI | 1 | 0.142 | 0.706 |  | 1 | 2.338 | 0.126 |  | Intra-brood sex-ratio | 1 | 0.093 | 0.760 |  | 1 | 0.013 | 0.910 |
| Social Group: Sex | 1 | 0.113 | 0.737 |  | 1 | 8.705 | **0.003** |  | BCI | 1 | 2.620 | 0.106 |  | 1 | 0.657 | 0.418 |
|  |  |  |  |  |  |  |  |  |  |  |  |  |  |  |  |  |
| *Call duration* |  |  |  |  |  |  |  |  | *Duration* |  |  |  |  |  |  |  |
| Call number | 1 | 0.313 | 0.576 |  | 1 | 6.631 | **0.010** |  | Social Group | 1 | 13.330 | **0.000** |  | 1 | 0.040 | 0.842 |
| Intra-brood sex-ratio | 1 | 0.076 | 0.783 |  | 1 | 2.190 | 0.139 |  | Call number | 1 | 7.974 | **0.005** |  | 1 | 0.199 | 0.656 |
| BCI | 1 | 1.629 | 0.202 |  | 1 | 0.462 | 0.497 |  | Intra-brood sex-ratio | 1 | 4.337 | 0.037 |  | 1 | 0.675 | 0.411 |
| Social Group: Sex | 1 | 1.460 | 0.227 |  | 1 | 7.640 | **0.006** |  | BCI | 1 | 3.701 | 0.054 |  | 1 | 0.151 | 0.698 |

**Figure EMS4: Modifications of begging calls’ median frequency, first quartile (Q25), standard deviation (Sd), and skewness of the frequency spectrum over the development of zebra finches reared either by zebra finches (ZFR) or by Bengalese finches (BFR).** Data points are mean (±se) when the corresponding subset of data contains several chicks, and mean only (without error bar) when the data point represents only one chick. The data set for one chick at a given age still represents multiple calls, so the corresponding data point is the mean on all these calls of the considered parameter. Grey shades are 95% Confidence Interval of the linear regression. Figures from results on post hoc models testing the group: Age interaction in males and females, the significance of the interaction between group and Age as a covariate is indicated in insert. * P≤0.05.


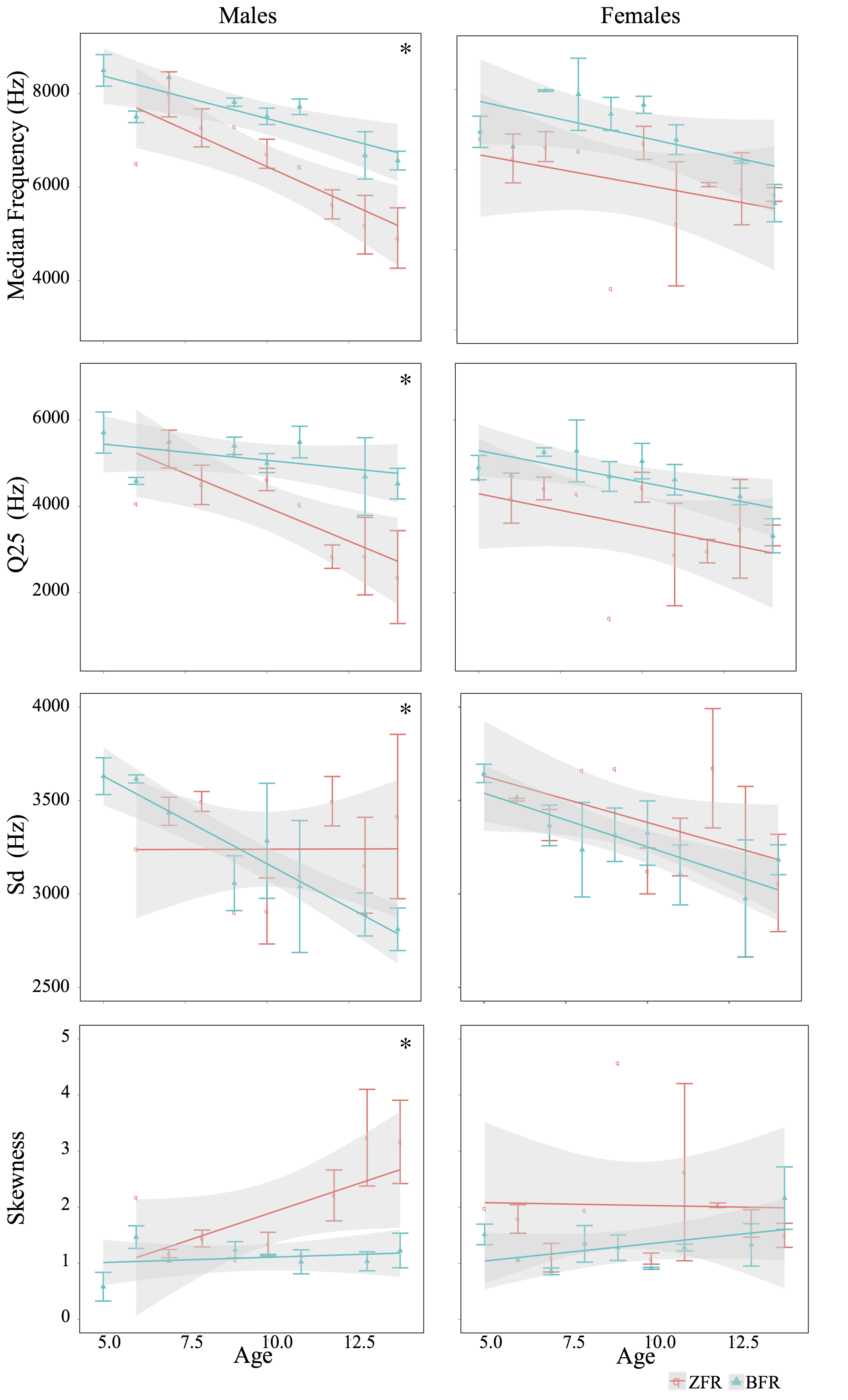


**Table ESM9: Statistical table for the analysis of chicks’ body condition at 14 DPH.** a- Results from the ‘drop1’ function of the model testing the main effects of the treatment without the non-significant interaction between sex and group. b- Results from the ‘drop1’ function (‘lmerTest’ R package) computed on the reduced data set (composed only of chicks for which we had recordings at 14 DPH exactly). ‘Drop1” computes all the single terms that can be added to or dropped from the model, fits those models and computes a table of the changes in fit. Only relevant interactions then compose the table. c-Estimates, standard errors and confidence intervals, generated with ‘lsmeans’ function (‘lmerTest” R package) of models on the complete and reduced dataset

|  | **Df** | **LRT** | **Pr(Chi)** |  |  |  |
| --- | --- | --- | --- | --- | --- | --- |
| **a-** |  |  |  |  |  |  |
| *BCI - additional model without the non-significant interaction between sex and group (51 subjects)* | | | | | | |
| group | 1 | 0.017 | 0.896 |  |  |  |
| sexe | 1 | 1.159 | 0.282 |  |  |  |
| Zday_14 | 1 | 0.353 | 0.552 |  |  |  |
| Intra-Brood sex-ratio | 1 | 0.089 | 0.766 |  |  |  |
| **b-** |  |  |  |  |  |  |
| *BCI - reduced dataset (23 subjects)* | | |  |  |  |  |
| Intra-Brood sex-ratio | 1 | 2.234 | 0.135 |  |  |  |
| Day of measurement (Day 14 ±1) | 1 | 1.210 | 0.271 |  |  |  |
| Group: Sex | 1 | 1.047 | 0.306 |  |  |  |
| **c-** |  |  |  |  |  |  |
|  | **Estimate** | **Standard Error** | **DF** | **t-value** | **Lower CI** | **Upper CI** |
| *BCI - all data (51 subjects)* | |  |  |  |  |  |
| ZFR Group | -0.078 | 0.195 | 26.400 | -0.400 | -0.478 | 0.323 |
| BFR Group | -0.044 | 0.472 | 13.400 | -0.090 | -1.061 | 0.972 |
| Females | -0.200 | 0.335 | 23.600 | -0.600 | -0.891 | 0.491 |
| Males | 0.078 | 0.260 | 17.800 | 0.300 | -0.469 | 0.626 |
| ZFR Group : Females | -0.310 | 0.335 | 15.300 | -0.930 | -1.022 | 0.402 |
| BFR Group: Females | -0.090 | 0.583 | 21.300 | -0.150 | -1.300 | 1.121 |
| ZFR Group: Males | 0.155 | 0.206 | 19.000 | 0.750 | -0.276 | 0.585 |
| BFR Group: Males | 0.002 | 0.474 | 13.300 | 0.000 | -1.020 | 1.023 |
|  |  |  |  |  |  |  |
| *BCI - reduced dataset (23 subjects)* | | |  |  |  |  |
| ZFR Group | -0.456 | 0.313 | 5.200 | -1.460 | -1.251 | 0.339 |
| BFR Group | -0.099 | 0.675 | 6.200 | -0.150 | -1.738 | 1.540 |
| Females | -0.227 | 0.485 | 11.100 | -0.470 | -1.292 | 0.839 |
| Males | -0.329 | 0.393 | 7.400 | -0.840 | -1.248 | 0.591 |
| ZFR Group : Females | -0.562 | 0.553 | 4.600 | -1.020 | -2.018 | 0.894 |
| BFR Group: Females | 0.109 | 0.802 | 9.900 | 0.140 | -1.680 | 1.897 |
| ZFR Group: Males | -0.351 | 0.304 | 1.600 | -1.150 | -1.996 | 1.294 |
| BFR Group: Males | -0.307 | 0.724 | 6.400 | -0.420 | -2.049 | 1.436 |

**Figure ESM5: Spectro-temporal features (spectra on the left column and spectrograms on the right column) of Bengalese finch begging calls at 14 (±1) DPH**. Six Bengalese finch nestlings (three males and three females) reared by three Bengalese finch pairs were recorded at several stages of their developpement using the same protocol as in the heterospecific cross fostering experiment (see methods), except that chicks were reared by their genetic parents. Here are presented three examples of calls from three different individuals. Note almost the same median frequency as in zebra finch begging calls of the same age but a much smaller spectral bandwidth. Bengalese finch calls are also more tonal than zebra finches’ ones (see spectrograms).


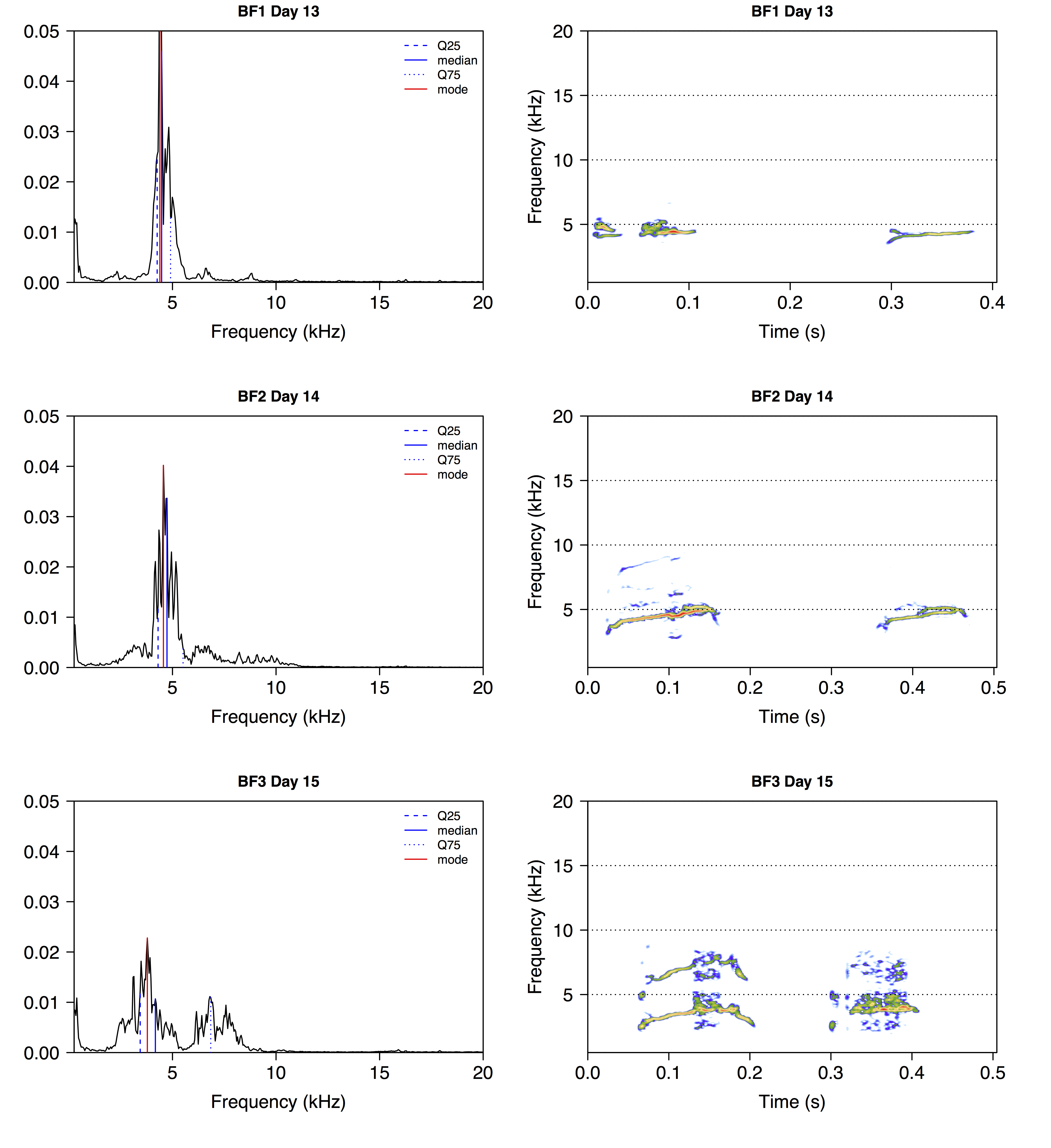


**Table ESM10: Comparison of begging call features of Bengalese finch chicks reared by Bengalese finch parents (BF-BFR), zebra finch chicks reared by Zebra finch parents (ZF-ZFR), and zebra finch chicks reared by Bengalese finch parents (ZF-BFR): step 1.** A Linear Discrinination Analysis (LDA) was computed on the two control groups (BF-BFR and ZF-ZFR) using 11 acoustic parameters. All nestlings were from 12 to 15 DPH. The first linear discriminant fonction (LD1) was then used to compare the acoustic structure of BF-BFR, ZF-ZFR and ZF-BFR (see Figure 5). The table gives the coefficients of LD1 and mean LD1 values of each parameter in each group (‘LDA.format’ function of ‘RVAideMemoire’ R package).

|  | **LD1 coefficients** | **Mean LD1 values** | |
| --- | --- | --- | --- |
|  |  | *BF-BFR* | *ZF-ZFR* |
| *Mean* | -0.099 | -0.622 | 0.180 |
| *Sd* | 0.765 | -1.150 | 0.332 |
| *Median* | 0.499 | -0.467 | 0.332 |
| *Mode* | 0.018 | 0.214 | -0.062 |
| *Q25* | 0.116 | 0.486 | -0.140 |
| *Q75* | -0.766 | -1.070 | 0.309 |
| *Skewness* | -0.667 | 1.147 | -0.331 |
| *Kurtosis* | 0.616 | 1.030 | -0.298 |
| *Sh* | 1.542 | -1.449 | 0.419 |
| *Duration* | -0.419 | 0.668 | -0.193 |
| *Ici* | -0.023 | 0.404 | -0.117 |

**Figure ESM6: Comparison of begging call features of Bengalese finch chicks reared by Bengalese finch parents (BF-BFR), zebra finch chicks reared by Zebra finch parents (ZF-ZFR), and zebra finch chicks reared by Bengalese finch parents (ZF-BFR): step 2.** BF-BFR: N=6 (three females, three males), ZF-ZFR: N=14 (seven females, seven males), ZF-BFR: N=15 (seven males and eight females). All nestlings were from 12 to 15 PDH. LD1 values for BF-BFR and ZF-ZFR calls resulted from the LDA in Table 13, LD1 values for ZF-BFR calls were calculated using the ‘predict’ function of ‘MASS’ R package.

Each point represents the mean (±SE) of the LD1 value of all individuals of a group. Normality and variance homogeneity of the data were not respected. Results of non parametric multiple comparisons following a significant Kruskal and Wallis test (‘kruskalmc’ function of ‘pgirmess’ R package): BF-BFR *vs.* ZF-ZFR: Observed diff._critical diff._=2308.7_144.8_, BF-BFR *vs.* ZF-BFR : Observed diff_critical diff_= 927.4_144.01_, ZF-ZFR *vs.* ZF-BFR Observed diff._critical diff._= 1381.3_96.1_. * = True difference found by the post hoc test.


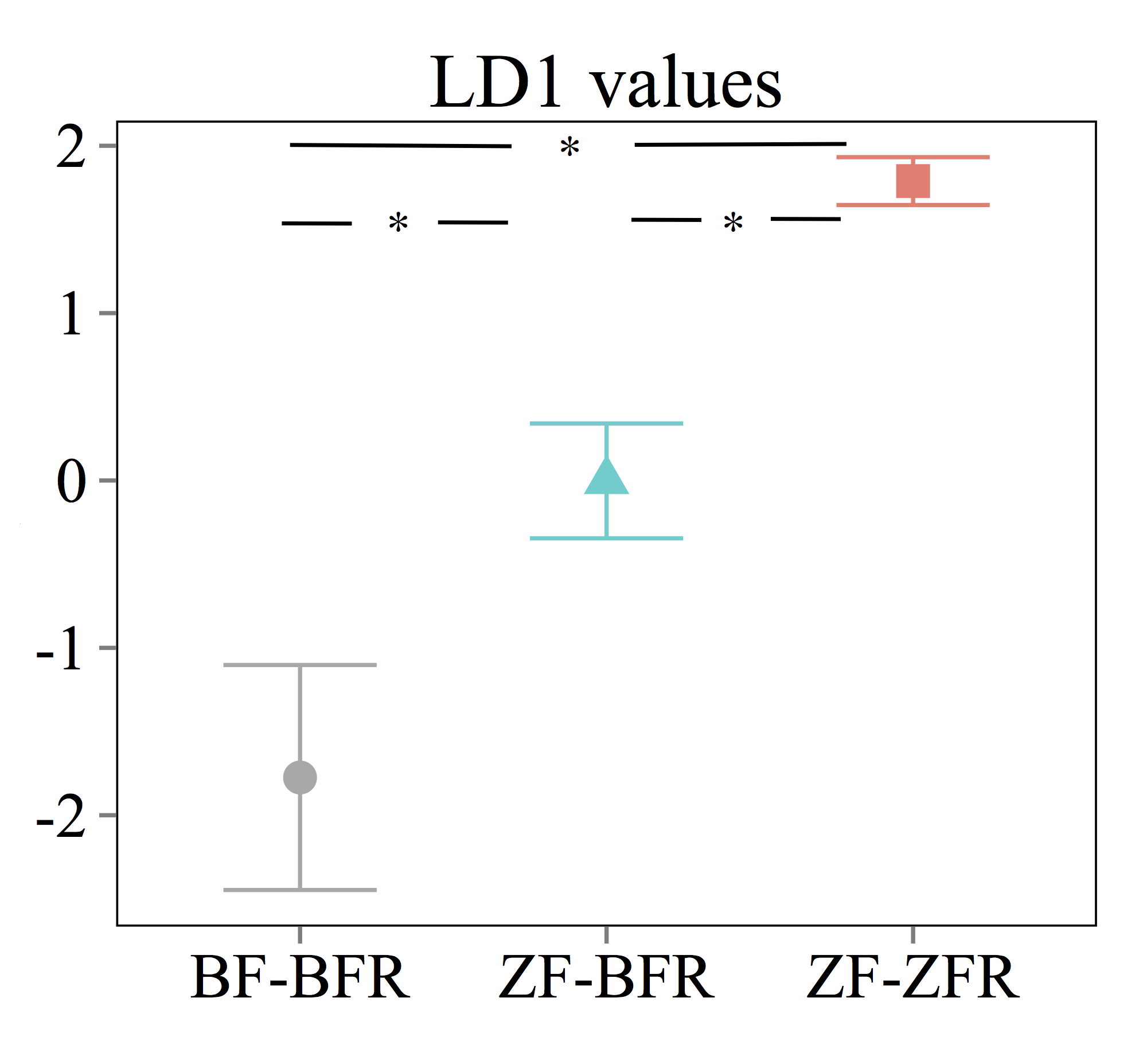

Supplement: Parental Influence on Begging Calls - ESM [file rsos150497supp1.doc]
